# Intranasal immunization of mice with chimera of *Salmonella* Typhi protein elicits protective intestinal immunity

**DOI:** 10.1038/s41541-024-00812-4

**Published:** 2024-02-06

**Authors:** Suparna Chakraborty, Pujarini Dutta, Ananda Pal, Swarnali Chakraborty, George Banik, Prolay Halder, Animesh Gope, Shin-ichi Miyoshi, Santasabuj Das

**Affiliations:** 1 Division of Clinical Medicine, ICMR- National Institute of Cholera and Enteric Diseases, P-33, C.I.T. Road, Scheme XM, Beliaghata, Kolkata, 700 010 India; 2 Department of Pediatrics, Steele Children’s Research Center, University of Arizona, Tuscon, AZ USA; 3 BD Biosciences, INDIA, Smart works Business Center, Victoria Park, 37/2 GN Block, Sector 5, Saltlake City, Kolkata, 700091 India; 4 Division of Bacteriology, ICMR- National Institute of Cholera and Enteric Diseases, P-33, C.I.T. Road, Scheme XM, Beliaghata, Kolkata, 700 010 India; 5 Graduate School of Medicine, Dentistry and Pharmaceutical Sciences, Okayama University, Okayama, Japan; 6 Collaborative Research Center of Okayama University for Infectious Diseases at Indian Council of Medical Research—National Institute of Cholera and Enteric Diseases, Kolkata, 700010 India; 7 ICMR-National Institute of Occupational Health, Meghaninagar, Ahmedabad, 3800016 Gujarat India

**Keywords:** Recombinant vaccine, Protein vaccines

## Abstract

Development of safe, highly effective and affordable enteric fever vaccines is a global health priority. Live, oral typhoid vaccines induce strong mucosal immunity and long-term protection, but safety remains a concern. In contrast, efficacy wears off rapidly for injectable, polysaccharide-based vaccines, which elicit poor mucosal response. We previously reported *Salmonella* Typhi outer membrane protein, T2544 as a potential candidate for bivalent (*S*. Typhi and *S*. Paratyphi A) vaccine development. Here, we show that intranasal immunization with a subunit vaccine (chimera of T2544 and cholera toxin B subunit) induced strong systemic and intestinal mucosal immunity and protection from *S*. Typhi challenge in a mouse model. CTB-T2544 augmented gut-homing receptor expression on lymphocytes that produced Th1 and Th17 cytokines, secretory IgA in stool that inhibited bacterial motility and epithelial attachment, antibody recall response and affinity maturation with increased number of follicular helper T cells and CD4+ central and effector memory cells.

## Introduction

*Salmonella enterica* serovar Typhi (*S*. Typhi), a Gram-negative, intracellular pathogen and the etiological agent of typhoid fever, is a global threat to public health. The organism spreads through the faeco-oral route, causing significant morbidities and mortalities in the developing countries, especially of the south-east Asian region. As per Global Burden of Disease data, 9.24 million new typhoid fever cases occurred worldwide in 2019 (females 4.17 million and males 5.07 million), leading to 110,000 deaths and 8.05 million (3.86–13.9) disability adjusted life years (DALYs). Incidences in males and females were 7.3 (4.5–11.1) and 8.6 (5.4–12.9), respectively per 100,000 populations^1^. After crossing the intestinal barrier, *Salmonella* Typhi, being carried by the infected macrophages, spreads to the distant body sites like the liver, spleen, bone marrow and lymph nodes. As an obligate intracellular pathogen, the bacterium creates a niche for replication within the phagosomes of the infected macrophages, rendering it difficult for the host to eliminate it from the body^2^. The problem of *S*. Typhi infection is further complicated by the emergence and spread of multi-drug resistant and extensively drug-resistant (XDR) strains across continents^3^. While the mortality in untreated typhoid fever reaches nearly 20%, an incompletely cured acute infection may be followed by an asymptomatic chronic carrier state, which poses an increased risk for the development of adenocarcinoma of the gall bladder^4^.

Improvement of sanitation, potable water supply and food hygiene is considered essential for elimination of *S*. Typhi. However, vaccination may play a significant role in decreasing the incidence in the endemic countries in the short run. Two vaccines, namely a live, attenuated *S*. Typhi Ty21a strain for oral administration and an injectable preparation, containing purified Vi polysaccharide (Vi-PS) are widely available commercially. Despite high seroconversion rates with good short term protection, they are at best modestly efficacious in the long run^5^. Oral typhoid vaccine induces systemic and intestinal secretory antibodies as well as good T cell response, but requires at least three to four doses for optimum protection. Further, it is not recommended for children below 6 years of age due to difficulty in swallowing large capsules of the vaccine formulation. Live typhoid vaccine has also raised safety concerns because of occasional reports of bacteremia^6^. To escalate immunogenicity with a single dose of vaccine administration, several research groups have developed alternative attenuated strains in the *S*. Typhi Ty2 background. Several mutant vaccine strains, such as Ty800 with the deletion of the global regulator phoP/phoQ, M01ZH09/ZH09 lacking aroC and ssaV genes and double mutant (aroC and aroD) strain CVD908 have passed Phase II trials and showed good mucosal and serum antibody response in the volunteers. However, CVD 908 resulted in vaccinemia with a single oral dose of 10^7^ viable organisms, and was later on modified by the deletion of another stress protein, htrA that prevented vaccinemia, but retained both humoral and cellular immune responses. To ensure more consistent serum anti-Vi antibodies, Vi was constitutively expressed in CVD 908 strain, generating CVD909. A prime boost regimen with orally administered CVD 909, followed by an injection of Vi-polysaccharide vaccine failed to induce strong Vi-specific antibody response, but instead, significantly raised Vi-specific IgA^+^ B memory cells. However, the need for pre-administration of buffer to neutralize stomach acid renders a potential delivery challenge for live attenuated vaccines^5^. None of the vaccine strains was shown to be effective against Paratyphoid infection.

Vi-polysaccharide vaccine, on the other hand, primarily works through the serum antibodies, but is poorly immunogenic with modest long-term efficacy, especially in children, because of T-cell-independent antibody response. Given the success of subunit vaccines for other bacterial infections, hunt for a highly efficacious typhoid vaccine, especially for smaller children (<2 years) that is safe and at the same time, capable of inducing durable protection is focused on developing typhoid conjugate (Vi-conjugate) vaccines (TCVs). A glycoconjugate vaccine (Vi-PS conjugated to tetanus toxoid, called Vi-TT), recently licensed in India has been reported to mount considerably high levels of durable protection through induction of memory B and T-cells^7,8^. The landmark TyVAC trial in Nepal, Malawi, and Bangladesh had shown protective efficacy of Typbar TCV to the extent of 79%, 84%, 85%, respectively^9–11^. However, protection conferred by Vi-TT and other TCVs, such as Vi-rEPA (polysaccharide conjugated to *Pseudomonas aeruginosa* exotoxin A), Vi-CRM197 and Vi-DT (diphtheria toxoid) would be exclusively mediated by the circulating anti-Vi antibodies due to the absence of intrinsic *Salmonella* proteins in these preparations^12^. Consequently, the emerging Vi-negative MDR (multidrug resistant) and XDR (extensive drug resistant) strains that were developed due to spontaneous genomic mutations in the regulatory regions of Vi polysaccharide of *S*. Typhi have evoked concerns^12^. There is a distinct threat that mass vaccination drive targeting Vi polysaccharide may exert a selection pressure for the existing Vi-negative *S*. Typhi and *S*. Paratyphi A and B strains, eventually rendering the vaccines to lose their protective efficacy. This has sparked an interest to generate future vaccines with universally present somatic antigens like T2544, which are immunogenic as well as essential for virulence of the bacteria. Antigen-specific, cytotoxic T cells, which were previously found to significantly augment anti-*S*. Typhi immunity through the elimination of intracellular bacteria were induced by recombinant T2544, as shown by our previous study, but not by the existing Vi conjugate vaccines^13^. Further, antibody secreting cells (ASCs), generated by Vi-PS and the TCVs will be primarily targeted to the systemic circulation, and not to the intestine^14^, whereas secretory antibodies, especially secretory IgA (sIgA) is believed to play a critical role in reducing systemic invasion of the luminal *S*. Typhi, thereby augmenting protection^15^.

Cholera toxin B subunit (CTB) has been extensively used as a vaccine adjuvant, especially for the protein antigens, owing to its nontoxic nature, ease of production of recombinant CTB and its fusion proteins in large quantities in *E. coli* and rapid renaturation of the recombinant proteins to the pentameric form of the native counterpart, enabling CTB to bind to its cognate GM1 ganglioside receptor^16,17^. This receptor is widely expressed by multiple, non-immune and immune cells (epithelial cells, macrophages, DCs and B cells), and CTB-binding to the receptor targets the antigens, co-administered or delivered as chimera to the desired cell populations, leading to augmented antigen uptake and presentation^16,17^. Other proposed mechanisms for the co-stimulatory effects of rCTB include direct lymphocyte activation^18^ and activation of the Toll-like receptors, although the identity of the TLRs remains unclear^19^. Adjuvanticity was most prominent when CTB was used as a genetically-fused partner of the vaccine antigen or chemically coupled to it, and delivered through the mucosal route. Indeed, CTB is one of the most potent mucosal adjuvants available to-date that attracted great attention^20,21^. Most antigens are poorly immunogenic when administered through the mucosal route, and tend to induce tolerance, especially at the intestinal mucosa. CTB often breaks the oral antigenic tolerance, but due to unknown reasons, significant oral adjuvanticity of CTB was only observed for live vaccines, such as oral cholera vaccine (Dukoral) that is already licensed for use^22^. In contrast, CTB administered through non-oral mucosal routes significantly enhanced antigen-specific humoral and cell-mediated immunity, not only at the local site, but also at distal mucosa, a phenomenon called “common mucosal immunity”^23^. Chemical and genetic conjugations of CTB with heterologous antigens from the simian immunodeficiency virus and *Schistosoma mansoni* have been proved to be potential vaccine candidates^24,25^. Adherent-invasive *Escherichia coli* (AIEC) tends to colonize the gut of patients with Crohn’s disease and exacerbate inflammation. Intranasal immunization with AIEC siderophore enterobactin, conjugated to CTB induced robust mucosal antibody response against the siderophore in the small intestine and colon, thus reducing gut colonization of AIEC and colitis severity^26^. However, an earlier study showed that intestinal mucosal antibodies elicited against siderophores after intranasal CTB-siderophore conjugate administration could significantly reduce *Salmonella* colonization of the inflamed gut, but failed to reduce inflammation^20^.

We had previously reported that an outer membrane protein of *S*. Typhi Ty2 strain (T2544) is strongly immunogenic in mouse and naturally-infected humans^27^. A candidate vaccine formulation based on T2544 induced high levels of serum antibodies as well as robust effector and memory T cell responses, when administered into mice through the subcutaneous route, and protected them against *S*. Typhi challenge^13,27^. T2544 was present in multiple clinical isolates of *S*. Typhi and *S*. Paratyphi, while T2544-specific antibodies were detected in the acute and convalescent sera of patients infected with *S*. Typhi^27^.

Here, we report that intranasal immunization with purified CTB-T2544 was also protective against oral *S*. Typhi infection. The chimeric antigen augmented (i) secretory IgA in stool which inhibited bacterial motility and epithelial attachment (ii) affinity maturation of antibodies with strong recall response, (iii) gut-homing receptor expression on lymphocytes that produced Th1 and Th17 cytokines, and (iv) the number of follicular helper T cells and CD4+ effector memory cells. These results suggested that CTB-T2544 is a promising candidate for the development of an intranasal vaccine against human *S*. Typhi infection.

## Results

### Cloning, expression and purification of recombinant CTB-T2544 chimeric protein

**Fig. 1:**
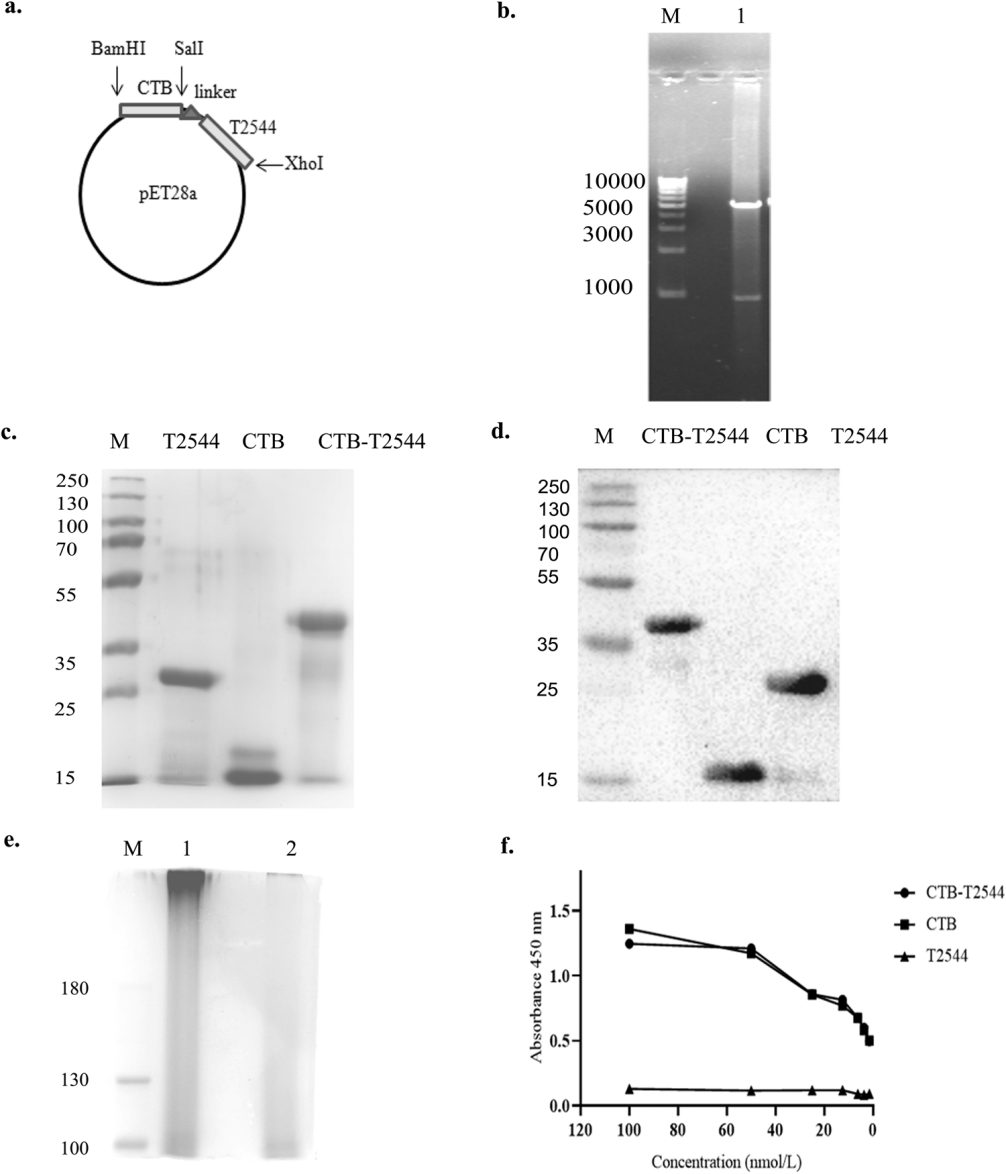
Purified antigen preparation and characterization. **a** Schematic diagram of cloned *ctx-b* and *t2544* genes in pET28a vector. **b** 1% Agarose gel electrophoresis of pET28a-ctb-t2544 after restriction digestion with BamHI and XhoI in Lane 1; Lane-M shows 1 Kb plus DNA ladder. **c** Recombinant purified proteins run in 10% SDS–PAGE, followed by Coomassie blue staining; Lane-M, pre-stained protein marker. **d** Western blot analysis of purified recombinant proteins; the blot was developed using anti-HIS monoclonal antibody; Lane-M, pre-stained protein marker. **e** Denatured (boiled) and non-denatured (un-boiled) rCTB-T2544 protein, resolved in 10% SDS PAGE, followed by Coomassie blue staining of the gel. Lane M, Pre-stained protein marker, Lane 1, unboiled rCTB-T2544; Lane 2, rCTB-T2544 after boiling. All the blots or gels were derived from the same experiment and were processed in parallel. **f** GM1-ELISA after coating the plates with GM1 ganglioside (1 μg/well), followed by the addition of purified proteins as indicated. Plates were developed using anti-HIS antiserum (1:2500).

To generate a gene chimera of *ctx-b* and *t2544*, 309 bp open reading frame (ORF) of *V.cholerae ctx-b* (cholera toxin B subunit) gene was amplified by PCR (Fig. S1a). In addition, ORF of *Salmonella* Typhi *t2544* gene, along with the coding sequence of glycine-proline-glycine-proline (GPGP) linker at its NH2-terminus (total size 663 bp) was PCR-amplified (Fig. S1b). The amplicon of the *ctx-b* gene was cloned into the prokaryotic expression plasmid pET28a, using BamHI and SalI restriction sites, followed by cloning of the 663 bp amplicon of linker-*t2544* at SalI and XhoI restriction sites immediately downstream of *ctx-b* (Fig. 1a). Confirmation of the fusion gene clone of *ctx-b* and *t2544* was done by colony PCR (Fig. S1c), restriction digestion (Fig. 1b) and finally nucleotide sequencing (Fig. S1d). To generate the recombinant chimeric protein, pET28a plasmid construct carrying the *ctx-b-t2544* fusion gene was transformed into the expression host *E.* *coli* BL21 (DE3) and the expression of the recombinant chimeric protein (rCTB-T2544) was induced by IPTG. rCTB-T2544 was purified by affinity chromatography using Ni-NTA agarose and the purity and specificity of the chimeric protein was confirmed by SDS-PAGE and western blots, respectively (Fig. 1c, d). Secondary structure of rCTB-T2544 derived from the analysis of the Far-UV circular dichroism (CD) spectra, using K2D2 (http://cbdm-01.zdv.uni-mainz.de/~andrade/k2d2/) indicated α-helical structure (72.38%) (Fig. S1e). Secondary structure of the other two proteins (rT2544 and rCTB) indicated 64.22% and 79.93% of α-helical structures, respectively. The yields of the purified rCTB-T2544, rCTB and rT2544 were 18.75 mg, 30 mg and 24 mg per liter of bacterial cultures, respectively. The endotoxin contaminations were found to be 0.015, 0.011, 0.008 EU/ml for rCTB, rT2544, and rCTB-T2544, respectively. Recombinant CTB-T2544, denatured by boiling, migrated in SDS-PAGE with the molecular weight marker that corresponded to the size of a chimera of T2544 and monomeric CTB (Fig. 1c**;** all the uncropped and unprocessed gel images are provided in Fig. S9**)**. However, the role of CTB as an adjuvant requires a pentamer that is capable to bind to its cognate cell surface receptor, GM1 ganglioside. The size of the non-denatured rCTB-T2544, resolved in SDS-PAGE corresponded to a higher molecular weight protein containing pentameric CTB (Fig. 1e). To check if the native chimera indeed contained a CTB pentamer, we studied by ELISA the binding of rCTB-T2544 to the GM1 ganglioside receptor. The results showed that both the recombinant CTB and CTB-T2544 were able to bind to the receptor in a dose-dependent manner, suggesting the existence of CTB pentamer (Fig. 1f).

### Immunization with rCTB-T2544 through the intranasal route protects against oral *S*. Typhi and *Vibrio cholerae* infections

**Fig. 2:**
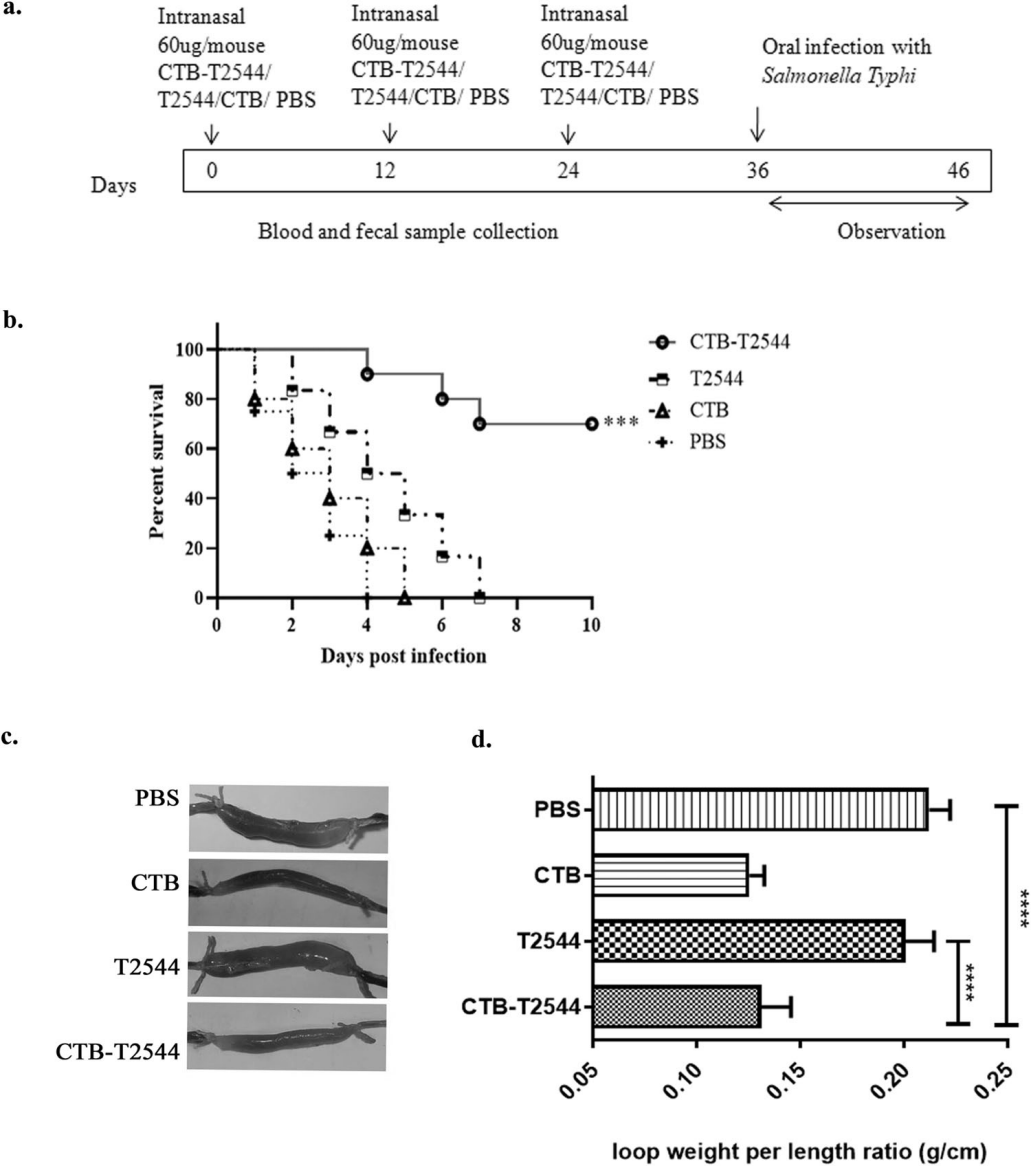
Protective efficacy of the recombinant antigens after intranasal immunization of mouse. **a** Immunization schedule, sample collection, and bacterial challenge of BALB/c mice. Blood and stool samples were collected before each immunization dose and 12 days after the last immunization. **b** Mouse survival assay. Kaplan-Meyer plot of cumulative mortality of mice immunized intranasally with the vehicle or the recombinant protein antigens. Mice (*n* = 10/group) were challenged with *S*. Typhi 12 days after the last immunization dose and were monitored for 10 days. Significance was calculated by comparing the survival of the mice immunized by rCTB-T2544 with T2544 by Log-Rank Mantel Cox test. ***p = 0.0001 **c** In parallel experiments, mice immunized as above were subjected to ileal loop assay by injecting 100 ng of cholera toxin into each loop. The animals (*n* = 8/group) were checked for fluid accumulation in the ileal loops after 8 hours. **d** Fluid accumulation was measured by calculating the weight divided by the length of individual loops. Significance was calculated by comparing the loop weight/ length ratio of CTB-T2544 group with the T2544 and PBS immunized group, using one-way ANOVA with *post hoc* analysis using Tukey’s multiple comparison test. Data represented as mean with SD; ****p value < 0.0001 vs. T2544 and PBS. Error bars represent SD.

The existence of the so-called “Common mucosal immune system”^23^ allows immunization through any mucosal route to induce immune response at the distant mucosal sites, albeit to a variable degree, due to migration of the activated immune cells. Intranasal immunization was earlier reported to elicit secretory IgA (sIgA) at the genital mucosa and salivary secretions^24–26^. To investigate if intranasal immunization with rCTB-T2544 induced protective immunity in the intestine, BALB/c mice were immunized with three doses of rCTB-T2544, rT2544, rCTB or the vehicle (PBS) at 12-day intervals (Fig. 2a). Twelve days after the 3rd dose, mice were challenged with 5 × 10^7 CFU (10 × LD_50_) of *S*. Typhi Ty2 strain through oral gavage (as mentioned under *Methods* section). An iron-overload mouse model of oral *S*. Typhi infection, established previously in our laboratory was used for the challenge experiment^27^. The results showed death of all the mice in the vehicle (PBS)-, rCTB- and rT2544- immunized groups within a period of 8 days, while 70% of mice that received CTB-T2544 were alive after 30 days of infection (Fig. 2b). Further, when the immunized animals were subjected to an ileal loop experiment with the administration of cholera toxin^28^, fluid accumulation in the loops was significantly less, resulting in lower loop weight/length ratio for rCTB- and rCTB-T2544-immunized animals compared with the ones that received T2544 or the vehicle (Fig. 2c, d). Together, the above results underscored the efficacy of rCTB-T2544 as a candidate bivalent vaccine for *S*. Typhi and *V*. *cholerae* infections.

### Intranasal immunization with rCTB-T2544 induces protective serum and mucosal antibody response

**Fig. 3:**
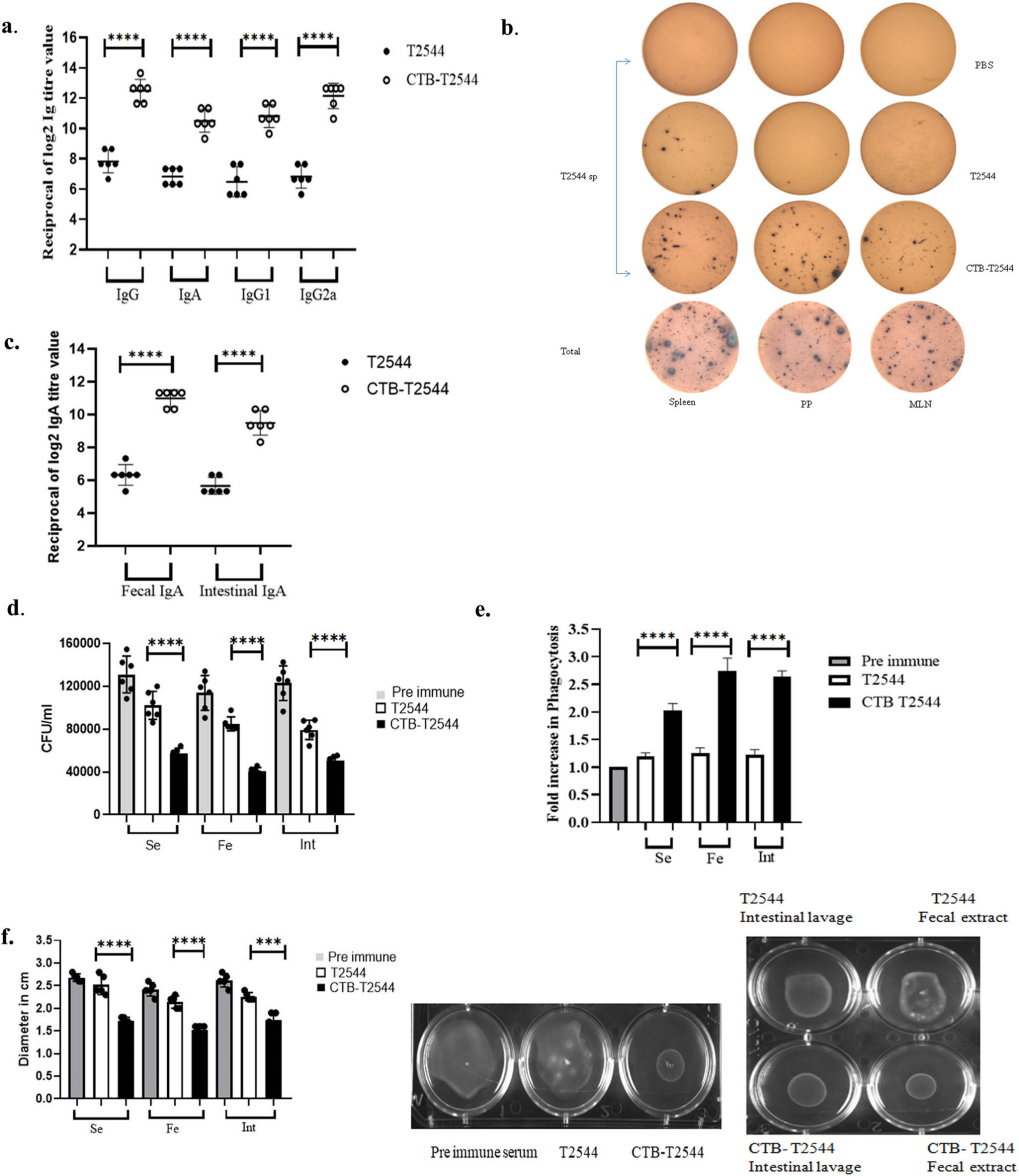
Intranasal immunization with rCTB-T2544 (*n* = 6) as above induces antigen-specific humoral and mucosal antibodies. **a** ELISA plates were coated with rT2544 protein and serum T2544-specific antibody titers were determined. Samples were serially half-diluted, from 1:200 to 1:25600 for IgG and 1:20 to 1:2560 for IgA. Optical density at 450 nm was recorded. The reciprocal of the titer, at which the absorbance of the sample was the same as the unimmunized mice samples was plotted in log2 scale. **b** Two weeks after the primary immunization, mice were sacrificed and cells were isolated from the spleen, MLN and Peyer’s Patches (PP). ELISPOT assay was performed with anti-mouse IgG, IgA and total immunoglobulin. **c** T2544-specific IgA antibodies in the fecal extracts and intestinal contents were quantified two weeks after the primary immunization. **d** Adhesion Inhibition Assay. Bacteria were preincubated with 1:50 dilution of the serum, fecal extracts or intestinal wash samples of the unimmunized and immunized mice for 30 min and used for adhesion assay as described under Methods. Mean CFU recovered from the lysed cells were plotted. **e** Opsonophagoctosis assay. Bacteria were pre-incubated with 1:50 dilution of the serum, fecal contents or intestinal lavage, followed by infection of the THP-1 cell monolayer. Opsonophagocytosis assay was performed as described under Methods. Phagocytosed bacteria were calculated after cell lysis and mean fold increase for the immunized samples compared with the pre-immune counterparts was plotted. **f** Motility inhibition assay. Motility of *Salmonella* Typhi Ty2 in LB agar (0.4%), containing 5% serum, fecal extract or intestinal contents from the immunized mice was measured by the zone diameter of bacterial growth after 6 h. The above experiments were repeated three times and mean of the values from all three experiments were plotted. Error bars represent SD. A representative picture from the motility assays is shown. Significance was calculated using two tailed unpaired *t* test, comparing CTB-T2544 group with the corresponding T2544 group. **** *p* < 0.0001, ***p = 0.0002.

To check if CTB could augment humoral immune response to the co-administered antigen T2544, antibody end-point titers (The reciprocal of the titer, at which the absorbance of the sample was the same as those from PBS-immunized mice) were determined by ELISA 12 days after completion of primary immunization dose. Markedly raised titers of T2544-specific serum IgG, IgG1, IgG2a, and IgA antibodies were observed in the mice immunized with rCTB-T2544, as compared with the rT2544-immunized mice (Fig. 3a). Similarly, CTB-specific IgG and IgA antibody titers were also raised in the serum (Fig. S3a). These results were in agreement with the recovery of substantial numbers of T2544-specific IgG and IgA antibody-secreting cells (ASCs) in the spleen, MLN, and Peyer’s Patches (PP) after intranasal rCTB-T2544, as analyzed by the enzyme-linked immunosorbent spot (ELISpot) assays, while ASCs were much fewer in the other groups of mice (Figs. 3b, S3b).

Intestinal sIgA antibody could significantly contribute to the protection against *S*. Typhi, an enteric pathogen. We measured antigen-specific sIgA titers in the stool and intestinal contents and found strong T2544-specific sIgA response only in the mice immunized with rCTB-T2544 (Fig. 3c), which also mounted CTB-specific sIgA (Fig. S3c). Protective efficacies of the serum and secretory antibodies were revealed by the inhibition of *S*. Typhi adherence to the intestinal epithelial cell monolayers, impairment of bacterial motility in the soft agar and the promotion of opsanophagocytosis. Sera, fecal extracts and intestinal contents from the experimental mice were pre-incubated with *S*. Typhi, which was subsequently used to infect HT-29 and THP-1 cell monolayers. Adhesion of the bacteria to HT-29 cell monolayer was studied by CFU counts and confocal microscopy. Bacterial adhesion to the cells was inhibited by the antibodies present in different samples, out of which maximum inhibition was found for the samples collected from rCTB-T2544 immunized mice (Figs. 3d, S3d). We also observed that a 1:5000 dilution of the rCTB-T2544 antibodies had comparable adhesion to 1:50 dilution of the pre-immunization samples, indicating that the former had 100-fold higher functional competence for adhesion inhibition (Fig. S6). Similarly, in the opsanophagocytosis assay, mean peak fold increase of bacterial phagocytosis by THP-1 cells was significantly higher following opsonization of the bacteria with antibodies from rCTB-T2544-immunized mice compared with the other groups of animals (Figs. 3e, S3e). Finally, we evaluated soft agar motility inhibition of *S*. Typhi after incubation with the above antibodies and found much greater inhibition when antibodies from the mice immunized with the chimeric protein were used (Fig. 3f). Together, the above results suggested that intranasal immunization with a CTB chimera of *S*. Typhi antigen (T2544) induced greater number of ASCs specific to the antigen in the secondary lymphoid tissues and higher levels of antigen-specific circulating and mucosal antibodies with greater protective efficacies against *S*. Typhi infection, when compared with the antigen administered without CTB. To further dissect relative contribution of the serum and mucosal antibodies, we performed adoptive transfer of pooled immune sera from rCTB-T2544-immunized mice to naïve mice, followed by a lethal bacterial challenge. This resulted in a 25% survival rate, suggesting a relatively minor role played by the serum antibodies in overall protection (Fig. S5a). To study the contribution from mucosal antibodies in the protection, we pre-incubated *S*. Typhi with intestinal lavage and fecal extracts from the immunized mice for 30 minutes before infecting the naïve mice. A lethal dose killed only 50% mice, while with a sublethal dose of the pre-incubated bacteria showed significant reduction in the colonization of the intestine (ileum, cecum, colon Fig. S5 b–d) and spread to the internal visceral organs (MLN, spleen and liver Fig. S5e–g) compared with the similar dose of bacteria that were not pre-incubated with mucosal antibodies. These findings highlighted a more significant role of mucosal antibodies in imparting protection.

### Intranasal immunization with rCTB-T2544 increased the recruitment of DCs and lymphocytes expressing gut-homing receptors to the MLN

**Fig. 4:**
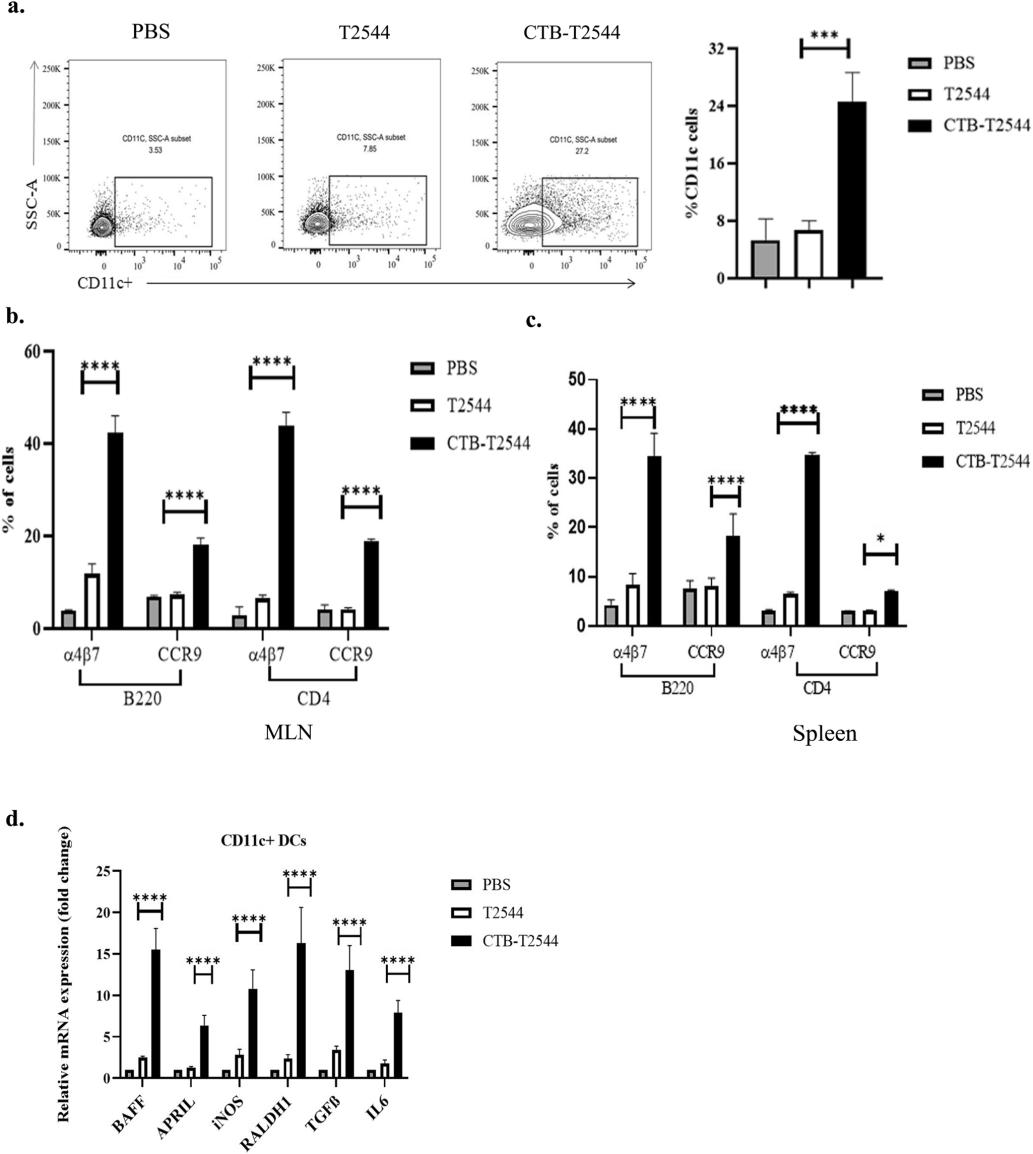
Intranasal immunization with CTB-T2544 directs DC and lymphocyte migration. **a** Twelve days after the primary immunization, MLN cells were surface-stained with CD11c and analyzed by flow cytometry. **b, c** Gut homing receptor expression on B and T lymphocytes. Twelve days after the primary immunization, lymphocytes isolated from the MLN (**b**) and spleen (**c**) of the experimental mice were stained with fluorochrome-tagged antibodies against cell lineage markers (CD4 and B220) and gut homing receptors (α4β7 and CCR9) and analyzed by flow cytometry. Data presented as percentages of cells out of the total T and B cells, expressing the homing receptors. **d** RT-qPCR analysis of gene expressions in DCs, isolated from the MLN of experimental mice were plotted. GAPDH-normalized expression of all the genes was represented as fold changes of the PBS-immunized group. All experiments were repeated three times, and data from a representative one are presented in (**a**) and mean ( ± SD) of the data from all three experiments are shown under (**b**–**d**). Significance was calculated using one way ANOVA (**a**) and two-way ANOVA (**b**,**c**) with *post hoc analysis using* Tukey’s multiple comparison test. Error bar represents SD. *p = 0.0281, ***p = 0.0008 and ****p < 0.0001 (T2544 vs. CTB-T2544).

Lung DCs were previously reported to imprint gut homing receptors on naïve (IgM^+^) B cells and CD4^+^ T cells in the local (mediastinal) lymph nodes after intranasal antigen administration, in addition to class switch recombination of B cells, generating IgG^+^ and IgA^+^ cells^29^. However, other studies had suggested that tissue homing of naïve lymphocytes is coupled with their site of activation by the DCs, and only the lymphocytes primed in the MLNs express α4β7 receptor that is required for migration to the intestine, although splenic lymphocytes show promiscuity with regard to tissue homing^30^. To address this issue in our study, we checked for DC recruitment to the MLNs of the immunized mice. The percentage of CD11c^+^ DCs in the MLNs after rCTB-T2544 immunization was nearly four times higher than after immunization with rT2544 (26.7% vs 7.0%) (Fig. 4a), indicating that intranasal CTB promotes DC migration to the draining nodes of the intestine. Next, to address DC migration, migratory DC (mDC) markers (MHC^hi^CD11C^int^CD103^+^) expression in the MLN DCs was assessed and found to be 3-fold higher (Fig. S7) in the MLN of the rCTB-T2544 immunized mice compared with the vehicle-immunized mice. Increased number of MLN DCs could result in greater number of lymphocytes expressing gut homing receptors. Indeed, we found significantly higher number of B and T cells, not only in the MLNs, but also from the spleen expressing α4β7 and CCR9 receptors (Figs. 4b, c, S4). DCs play a critical role in mucosal sIgA production by expressing several factors like BAFF, APRIL, iNOS, RALDH1, TGF-β and IL-6^31^. Given increased DC migration to the MLNs and strong sIgA response in the intestine after intranasal immunization with CTB-T2544, we studied for the IgA-inducing factor expression by the MLN DCs. The results showed augmented expression of the factors as above by the MLN DCs (Fig. 4d). Together the above results suggested that intranasal CTB upregulates the expression of IgA-inducing factors on the local DCs and promotes their migration to the MLNs. These MLN DCs imprint gut homing receptors on the lymphocytes, which then migrate to the intestinal mucosa to execute the effector function by sIgA and cytokines production.

### CTB-T2544 immunization induces a balanced T helper cell response

**Fig. 5:**
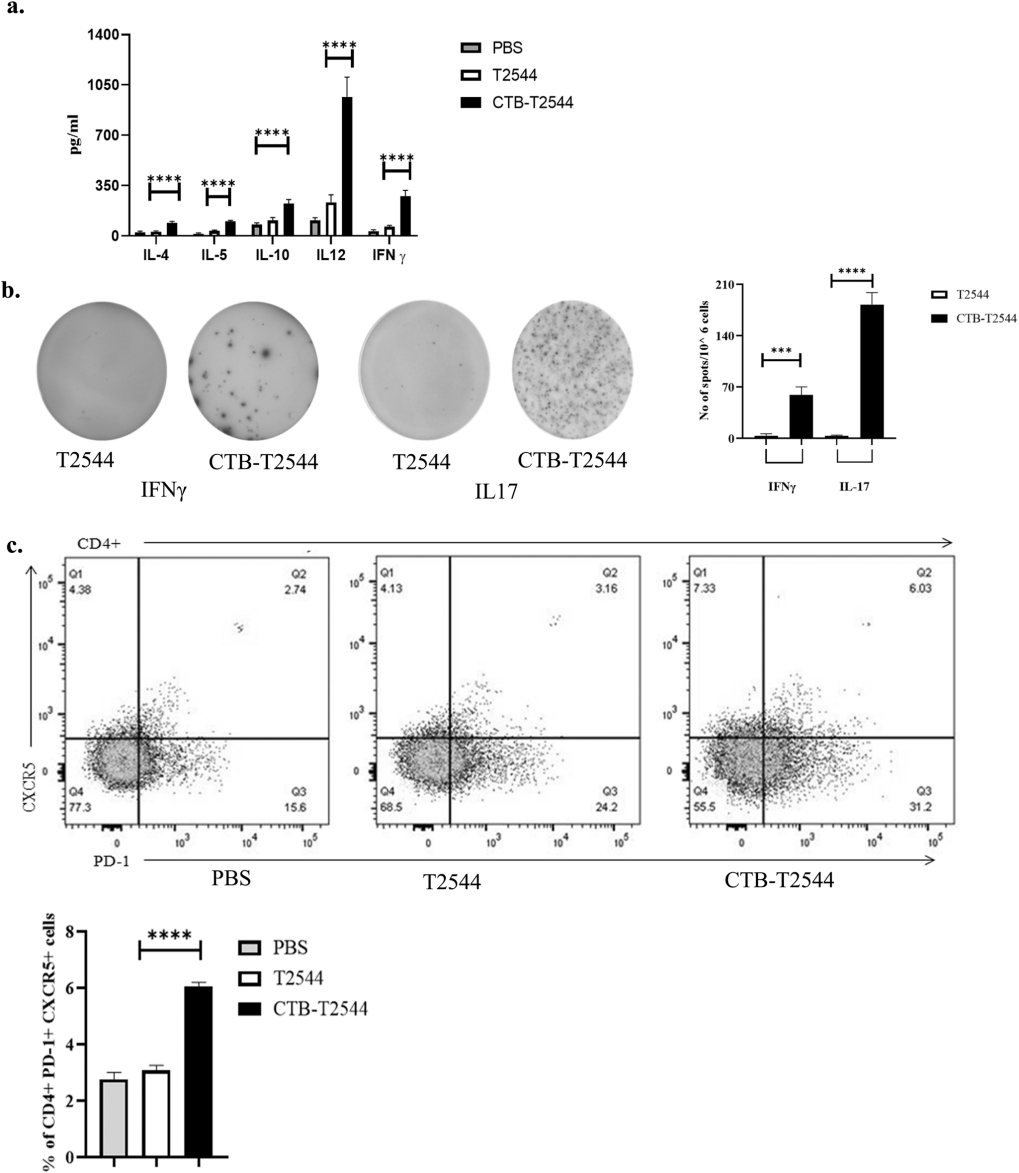
T cell response measured after 12 days of primary immunization. **a** Th1 (IL-12, IFN-γ), Th2 (IL-4, IL-5), and Treg (IL-10) cell cytokines in the serum samples of the unimmunized and immunized mice were measured by ELISA. **b** Cells from the PP of the experimental mice were incubated with 10 µg of T2544 for 24 h. Cytokine-secreting cells were enumerated by ELISpot assays and data presented as spot forming cells/10^6^ total cells analyzed. **c** Analysis of T_FH_ (T follicular helper) cells in the MLN. Cells were detected by measuring the expression of CXCR5 and PD-1 on CD4^+^ cells using flow cytometer. Significance was calculated by one way (**b**, **c**) and two-way (**a**) ANOVA with *post hoc* analysis using Tukey’s multiple comparison test. The experiment was repeated three times and representative images from one experiment are shown. Bar diagram represents the statistical data from all three experiments. Error bars represent SD. ****p < 0.0001, ***p = 0.0006.

Th1 response is required for the clearance of intracellular pathogens like *Salmonella*^32^, while Th2 and Th17 cells promote serum antibodies and sIgA production, respectively. We found significantly elevated, circulating Th1 (IL12 and IFNγ) and Th2 (IL-4, IL-5, IL-10) cytokines in rCTB-T2544-immunized mice as opposed to only modest elevation in the comparator immunized groups (Fig. 5a). In addition, the number of IFNγ- and IL-17A-secreting CD4^+^ T cells (Th1 and Th17 cells, respectively) in the Peyer’s Patches was also increased markedly after CTB-T2544 immunization (Fig. 5b). We also checked if CTB induced follicular helper T (T_FH_) cells, which play a crucial role in the germinal center formation and the development of high-affinity antibodies and memory B cells^33^. T_FH_ cell number in MLN after immunization with rCTB-T2544 was doubled compared with rT2544 or no immunization (6.03% vs 3.06% vs 2.74%, respectively) (Fig. 5c). Together, these results suggested that rCTB-T2544 chimera helped to create a protective milieu in the intestine through the induction of T cell subsets and cytokines.

### CTB-T2544 immunization elicits memory response

**Fig. 6:**
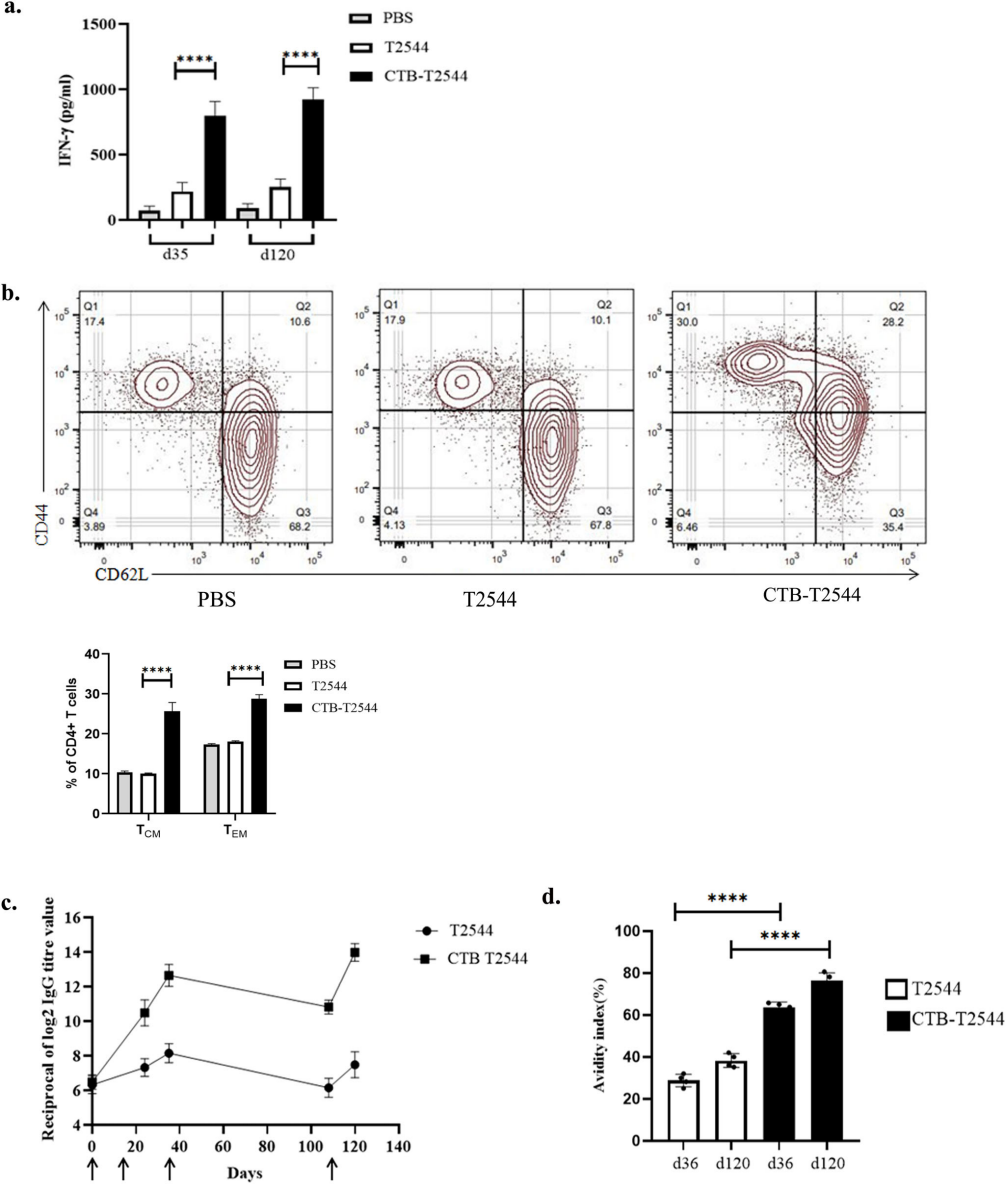
Immunological memory response. **a** Memory T cells were analyzed by ELISA for IFN-γ release in the culture supernatants of CD4^+^ T cells, isolated from the spleen of the experimental mice and co-cultured with BMDCs, pulsed with rT2544. Data represents mean( ± SD) of three independent experiments. **b** The percentages of T_EM_ (CD4^+^CD44^hi^CD62L^lo^) and T_CM_ (CD4^+^CD44^hi^CD62L^hi^) cells from the spleens of different experimental groups were quantified using flow cytometry. Representative images from one of the experiments and statistical analysis of the data from all three experiments are shown. **c** Serum antibody titers (mean ± SD) measured in the immunized mice (*n* = 6) at the indicated time points are shown. Arrows indicate primary (3 doses at 0, 12 and 24th day) and booster (108th day) immunization. Mean ± SD were plotted for CTB-T2544 and T2544. **d** Avidity assay. Anti-T2544 avidity index was measured by ELISA and represented as the ratio of IgG bound to T2544 in the presence and absence of 4 M urea, multiplied by 100. Significance was calculated using one way (**a**, **d**) and two-way (**b**) ANOVA with *post hoc* analysis using Tukey’s multiple comparison test. Error bars represent SD. ****p < 0.0001.

Antigen-specific memory T cell response was evaluated by co-culture of the CD4^+^ T-cells, isolated from the spleen of the experimental mice with *S*. Typhi-pulsed bone marrow dendritic cells (BMDCs) derived from naïve mice. IFNγ release measured in the culture supernatants was significantly higher for the CD4^+^ cells recovered from the mice immunized with rCTB-T2544 as compared with the animals that received rT2544 or left unimmunized, suggesting augmentation of the memory response by CTB (Fig. 6a). With respect to specific memory cell subsets, percentages of both central memory (CD62L^high^CD44^high^) and effector memory (CD62L^low^CD44^high^) T cells (T_CM_ and T_EM_)^34^ were increased after immunization with the chimeric antigen containing CTB (Fig. 6b). In contrast, these mice had half the number of naïve CD4^+^ cells in the spleen as compared with the other immunized groups. To compare the induction of memory B cells after immunization, a booster dose was administered on 108th day of experiment and serum anti-T2544 antibodies were measured 12 days later. A steeper rise of antibody production with significantly higher titers was observed after rCTB-T2544 immunization as compared with the immunization using rT2544 alone (Fig. 6c), suggesting the critical role played by CTB in antigen-induced differentiation of memory B cells into the plasma cells, producing IgG. Avidity index of anti-T2544 IgG antibodies was assessed by measuring the OD of the ELISA plates after washing the immune complexes with or without 4 M urea-containing buffer. We observed significantly high avidity index (60–65%) after the primary immunization, which further went up to 70–78% following the booster immunization (Fig. 6d), suggesting successful priming. In contrast, intranasal T2544 immunization resulted in poor priming (avidity index < 35%). These results together suggest that intranasal rCTB-T2544 may elicit long-term immunity against *S*. Typhi infection.

## Discussion

Typhoid is a systemic febrile illness, caused by an intracellular pathogen, acquired through the intestine. Protective immunity following typhoid occurs in minority of the cases^35^. This correlates with rather modest immune response after oral immunization with live Ty21a vaccine and the requirement of multiple doses^5^. In contrast, injectable TCVs, especially Vi-TT demonstrated a high level of durable protection in clinical trials, but an attack rate of 35% was observed in a CHIM (Controlled Human Infection Model) studies. However, several challenges related to the TCVs still persist that merit immediate attention. Available studies suggest that serum antibodies may not be sufficient for protection, since patients recovered from typhoid fever remain susceptible to re-infection despite elevated levels of circulating antibodies against multiple *S*. Typhi antigens (H, O, Vi-PS, porins etc)^13^. High anti-Vi IgG titers were also present in the chronic biliary carriers from the endemic populations^36^. This perhaps underscores a critical role played by the mucosal and cell-mediated immunity for overall protection against typhoid infection. Parenteral vaccination is considered a poor method for inducing mucosal immune response that was previously reported to confer protection against cholera and typhoid fever^17^. A mucosal vaccine will be highly desirable also for the ease of administration without technical assistance. Further, mouse infection studies found that *Salmonella*-specific T cells were required for late clearance of intracellular bacteria^37^. This suggests that no single component of the immune response is sufficient to provide complete protection against *S*. Typhi. We had earlier shown that T2544 induces antigen-specific cytotoxic T lymphocytes (CTLs) in mice in addition to serum antibodies^13^. In the present study, we demonstrated that a strong gut mucosal immune response in the form of protective secretory antibodies (sIgA) and gut homing lymphocytes (ASCs and Th1/Th17 cells), induced by the CTB-T2544 chimera may significantly contribute to the protection of mouse against oral *S*. Typhi challenge. The tested vaccination regimen for CTB-T2544 in mice had three intranasal doses, as opposed to single intramuscular (IM) dose of TCVs in humans. It is usual to administer multiple doses of candidate vaccines for animal studies, while single dose regimens of these formulations are often developed during clinical trials^38–40^. Mucosal immunity following CTB-T2544 will increase bacterial shedding and reduce their stay inside the body. This might provide herd protection, since *S*. Typhi does not survive long outside the human system. Herd protection was not observed with Vi-TT in the clinical trials conducted so far^11^. Another concern related to TCV, especially for low- and middle-income countries, is its relatively high cost of production, because of the requirement of complex chemical conjugations and significant batch-to-batch variations. In contrast, bacterial expression of recombinant CTB-T2544 will substantially reduce the cost and eliminate batch variations. Finally, like Vi-TT, CTB-T2544 is expected to be compatible with other vaccines, given that T2544 is absent outside the genus *Salmonella*. Because CTB functions as adjuvants to the co-administered antigens as well, CTB-T2544 may augment immunogenicity of other childhood vaccines.

CTB-T2544 chimeric antigen was generated by genetic fusion of *t2544* to the C-terminus of *ctx-b* gene, using a non-furin (GPGP) linker sequence^41^. CTB promotes immune response to both the co-administered antigens and antigens delivered as CTB chimera, but the chimeric formulations are perhaps more immunogenic, since conjugated CTB elicited 10-fold higher immunogenicity to ovalbumin than co-administered CTB^42^. Also, CTB C-terminus fusion led to the best pentamer formation, with strongest binding affinity to the GM1 receptor^16^. We observed similar binding for CTB-T2544 and CTB pentamer to the GM1 receptor, indicating that pentameric structure of CTB was retained in the chimera. However, we did not observe significantly lower expression levels of CTB-T2544 compared with CTB alone, as was reported earlier for recombinant CTB chimera.

CTB showed efficacy as vaccine adjuvant after administration through multiple routes, most notably for oral and nasal vaccines. While oral adjuvanticity was largely limited to the live vaccines^43^, nasal administration worked well for subunit vaccines as well^16^. This was proposed to be mediated by increased DC activation and T and B cell responses, leading to five to tenfold reduction in antigenic dose requirement. For example, 10 μg DNA co-administered with CTB induced cellular immune responses to the magnitude of 50 μg DNA used alone in the IFN-γ ELISPOT assay^19^. In the present study, T2544 conferred over 70% protection to mice against *S*. Typhi challenge (10 × LD_50_ dose) when administered as CTB chimera, but immunization with a 3-fold higher dose of T2544 alone was non-protective. CTB was shown previously to activate TLR signaling in BMDCs, resulting in pro-inflammatory cytokine production^19^. In vivo and ex-vivo experiments with subcutaneous KLH immunization showed that CTB can also function as a potent adjuvant through direct stimulation of antigen-primed CD4^+^ and CD8^+^ T cells and stimulates IL-2, IL-4, and IFN-γ production^18^. However, most investigators had reported a Th2-predominant immune response with CTB and tolerization to the co-administered antigens in vivo, while either a Th1 or a more balanced T-helper (Th1/Th2) and T-regulatory (Treg) response was observed by others^16,44,45^. CTB-T2544 also displayed a mixed T cell response (both Th1/pro-inflammatory and Th-2/anti-inflammatory cytokines) (Fig. 5a, b). Published literature and the current study suggest that the nature of the immune response might depend on multiple factors, including the antigen type, dose and route of administration.

Efficacy of typhoid vaccines is traditionally monitored by serum antibodies, especially antigen-specific IgG titers. CTB significantly raised the titers of both IgG1 and IgG2a specific to T2544 in the serum (Fig. 3a), indicating humoral as well as cell-mediated immune responses. However, TCV under TyVAC trial failed to establish protective IgG and IgA titers despite inducing high levels of durable protection. In the controlled human infection model (CHIM) studies, serum IgG and SBA titers at the time of challenge after vaccination with Vi-PS or Vi-TT poorly matched with the protection against *S*. Typhi^46^. Instead, serum IgA titers showed significant correlation with Vi-PS-induced protection^47^, underscoring potential role for intestinal sIgA. Stool sIgA titer is also believed to correlate with protection conferred by oral vaccines^48^. However, direct demonstration of protection by sIgA for typhoid vaccines is lacking in the literature. We reported here high sIgA titers, along with increased number of IgA ASCs in the intestine of mice after intranasal CTB-T2544 immunization (Fig. 3b). Further, we demonstrated functionality of the secretory antibodies by in vitro assays, such as adhesion and motility inhibition and their in vivo protective role by infecting mice with *S*. Typhi, preincubated with fecal extracts and intestinal washes (Figs. 3, S5). The above results also suggest that intranasal CTB-T2544 may potentially confer herd protection to the vaccinated community by increasing bacterial shedding from the intestine, which was not found with TCVs^11,49^.

Our study found marked increase in the percentages of B cells, expressing gut homing receptors (α4β7 and CCR9) in the mouse MLNs and spleen (Fig. 4b, c). Earlier reports showed that intranasal immunization with inactivated CT holotoxin stimulates lung conventional DCs (cDCs) to promote IgA class switch of B cells in the regional lymph nodes and imprint α4β7 and CCR9 on IgA ASCs, resulting in their migration to the intestine^29^. However, we found significant expansion of CD11c^+^ DCs, expressing IgA-inducing factors in the MLNs, which was not described earlier after intranasal immunization (Fig. 4d). This coupled with gut homing receptor expression on MLN B cells and increased number of IgA^+^ ASCs in the Peyer’s Patches, along with sIgA in the intestine suggests that CTB might have induced DC migration from the lungs to the MLN where they orchestrated B cell class switch and gut homing of IgA^+^ B cells^31^. To address DC migration, we checked for migratory DC (mDC) markers (MHC^hi^CD11C^int^CD103^+^) expression in the MLN DCs. The results showed threefold higher number of mDCs in the MLN (Fig. S7) in the mice immunized with CTB-T2544 compared with the vehicle-immunized animals. However, we did not rule out gut-homing lymphocyte generation in the lung-draining lymph nodes as well. Although in vitro experiments conducted previously suggested that CTB might require LPS to stimulate dendritic cells to promote IgA production by B cells^50^, recombinant CTB-T2544 contained negligible amounts of LPS.

Memory B (B_M_) cells are required for long term protection against typhoid fever. A strong association between the antigen-specific B_M_ cells and antibody titers were reported in the literature^33^. Several studies had reported significantly higher antigen-specific serum antibody response with chimeric CTB compared with CTB co-administration with the antigens^42,51^. In contrast, a separate study suggested weaker adjuvant function of CT or CTB, conjugated to streptococcal surface protein antigen AgI/II than to simultaneous, but separate administration with the antigen for recall antibody response to booster intranasal immunization^52^. Moreover, booster dose only enhanced AgI/II specific IgM and IgA, but not IgG. In contrast, we observed strong adjuvant effects of CTB for both the primary and booster immunization with robust IgG induction (Fig. 6c). The difference in the outcome of CTB adjuvanticity between the above two studies might be due to the fact that the authors from the earlier report used chemical conjugation of CTB and Streptococcal antigens as opposed to genetic fusion of CTB and T2544 in our study. T_FH_ cells were reported to support B_M_ cell generation and T cell-dependent affinity maturation of antibodies, resulting in higher avidity secondary antibodies. This was corroborated by our results that showed doubling of the T_FH_ cell number in the MLN and significantly increased avidity of anti-T2544 antibodies after a recall response (Figs. 5c, 6d).

In addition to B cells, large proportion of CD4^+^ T cells from the spleen and MLNs expressed gut homing receptors. Imprinting of homing receptors on T cells by lung DCs was reported previously following intranasal immunization with CTB^53^. Intestinal CD4^+^ cells in our study expressed Th1 and Th17 cytokines, both of which are critical for clearing of the intracellular bacteria, while Th17 also promotes sIgA production^17,54^. A previous study with human volunteers who received oral Ty21a vaccine showed that IL-17A, predominantly produced by the CD8^+^ T cells in the peripheral circulation, contributes to mucosal protection against *S*. Typhi infection^55^.

Circulating as well as tissue-resident memory T cells provide protection against *S*. Typhi. Oral vaccination of volunteers with Ty21a generated largely monofunctional CD4^+^ T_EM_ or T_EMRA_ (T Effector Memory Re-expressing CD45RA) cells that produced high levels of IL-17A or TNF-α. In contrast, CD8^+^ T cells were predominantly polyfunctional, 80% of which showed T_EM_ phenotype and produced triple cytokines^56^. The requirement of circulating memory CD4^+^ and CD8^+^ T cells as well as TNF-α and IFN-γ for protection against *Salmonella* was shown in mice after oral immunization with aroA-attenuated live vaccine strain or intradermal immunization with porins^57,58^. Here, we show that mice that received intranasal CTB as a chimeric partner augmented T2544-specific memory CD4^+^ response, which upregulated IFN-γ production and were constituted by equal numbers of T_EM_ and T_CM_ (Fig. 6a, b).

There are some limitations of the current study. Immunogenicity and protective efficacy of CTB as an antigen was revealed by the induction of CTB-specific serum and mucosal antibodies in the mice immunized with CTB-T2544 as well as markedly reduced fluid accumulation in the ileal loops after challenge with cholera toxin. Previous studies with intranasal immunization with rCTB conferred 70% protection to rabbits against *V. cholerae* challenge, while co-administration of purified CTB and TcpA was 100% protective^59^. Another study showed intranasal, intraperitoneal, and subcutaneous immunization of mothers with rCTB alone provided high level protection to the pups against lethal challenge with *Vibrio*^60^. However, further studies are needed to evaluate how CTB-T2544 performs vis-à-vis other cholera vaccines and if the former can boost immune response to other vaccines against *V*. *cholerae* infection.

The present study suggested a higher relative contribution from the intestinal secretory antibodies for protection against *S*. Typhi following nasal immunization with CTB-T2544 (Fig. S5). This question may be further addressed in future studies by infecting immunized, polymeric immunoglobulin receptor (pIgR) knock-out (pIgR^−/−^) mice, which will be devoid of sIgA from the intestinal secretions. Studies may also be designed by comparing different routes of immunization and/or their combinations to establish the immune correlates of protection against *S*. Typhi infection after CTB-T2544. Such studies could be useful to formulate a prime-boost regimen (intranasal priming and boosting through other routes) with our candidate vaccine. Another important aspect of future studies could be the vaccine efficacy against MDR and XDR strains of *S*. Typhi.

Given that mucosal vaccines are more desirable than the injectable preparations for mucosal infections and polysaccharides have limited immunogenicity as mucosal antigens, further investigations need to be carried out to exploit the conserved, immunogenic protein antigens of *Salmonella* for the development of highly efficacious mucosal vaccines. Another desirable goal is to design multivalent vaccines that can simultaneously protect against typhoidal and non-typhoidal *Salmonella* infections, since no vaccines are currently available against *S*. Paratyphi and NTS. We have developed a multivalent vaccine containing T2544 and O-specific polysaccharide of *S*. Typhimurium that confers protection against *Salmonella* Typhi/Paratphi/Typhimurium with cross protection against *S*. Enteritidis [61].

Overall, we studied the adjuvant function of CTB to the protein subunit vaccine candidate T2544 for intestinal mucosal immune response after immunization through the intranasal route. Recombinant CTB-T2544 chimera induced high titers of antigen-specific and protective serum IgG and mucosal sIgA antibodies. sIgA response was mediated by CTB-elicited migration of DCs, expressing IgA-inducing factors to the MLN and imprinting of gut homing receptors on the lymphocytes over there. This study also highlighted the role of CTB in inducing a balanced T helper cell response, including the Th1, Th2, Th17, and T_FH_ cells. This, accompanied by augmented, antigen-specific memory B and T cell response after intranasal CTB elicited a strong and durable mucosal immunity at the intestine, leading to protection against oral *S*. Typhi infection.

## Methods

### Cells, strains, growth condition, and plasmid

HT-29 and THP-1 cells used in this study were purchased from the American Type Culture Collection (ATCC, UDA). HT-29 cells were maintained in Dulbecco’s modified Eagle’s medium, supplemented with 10% fetal bovine serum (FBS). THP-1 cells were grown in RPMI 1640, supplemented with 10% FBS and 0.05 mM 2-mercapto ethanol. *Salmonella* Typhi Ty2, a gift from J. Parkhill (Sanger Institute, Hinxton, UK) was grown in Hektoen enteric agar (BD Difco). *Escherichia coli* strain BL21, a kind gift from Dr. Rupak k. Bhadra (Indian Institute of Chemical Biology) was maintained in Luria–Bertani agar at 37 °C. Liquid cultures of the bacterial strains were grown in Luria-Bertani broth (BD Difco). pET-28a was purchased from Addgene, USA.

### Cloning, expression, and purification of recombinant rCTB-T2544 protein

A 309 bp coding sequence of *ctx-b* gene was PCR amplified (CTB fus Prot FP: CGCGGATCCACACCTCAAAATATTACTGATTTGTG; CTB fus Prot RP: GCCGTCGACATTTGCCATACTAATTGCGGC) from *V. Cholerae* and cloned in pET28a vector between BamHI and SalI restriction sites. Later on, *t2544* along with an upstream linker sequence (*ggaccaggacca* is the gene codon of a non-furin linker containing glycine and proline amino acids) (total sequence length 663 bp) was PCR amplified and the PCR product was cloned between SalI and XhoI restriction enzymes of pET28a-CTB plasmid (Fus T2544 FP: GCCGTCGACGGACCAGGACCAGAAGGGATCTATATCACCGGG; Fus T2544 RP: GCCCTCGAGTTAAAAGGCGTAAGTAATGCCGAG). The newly generated construct was transformed into the expression host *E. coli* BL21 (DE3) and the recombinant fusion protein (rCTB-T2544) expression was induced with 1 mM Isopropyl β-D-1-thiogalactopyranoside (IPTG, SIGMA) by culturing the bacteria in LB medium at 37 °C. rCTB-T2544 was extracted as an insoluble protein from inclusion bodies by sonication and solubilized by denaturing with 8 M urea. Denatured protein was purified by affinity chromatography using Ni^2+^-conjugated agarose (Qiagen) and then renatured by gradual removal of urea by dialysis using a 10 kD membrane. Purified protein was quantified by Bradford Reagent and the purity was confirmed by SDS-PAGE, stained with Coomassie Brilliant Blue.

### Immunoblot and pentamer analysis assay

Aliquotes of the protein samples were subjected to SDS-PAGE and transferred to PVDF membrane (Millipore). After blocking with 5% BSA in PBS containing 0.05% Tween 20 for 1 h, membranes were incubated with anti-His antiserum (1:2500 dilution) overnight at 4 °C. After washing, the membrane was incubated with HRP-conjugated anti-rabbit IgG (1:10,000) for another 1 h. Then, the membrane was developed by adding chemiluminescent substrates (SuperSignal West Pico, Thermo Scientific) using ChemiDoc.

To assess whether CTB-T2544 folds into pentameric state during SDS-PAGE, samples were subjected to non-denaturing and denaturing conditions. In the former conditions, samples were not boiled, and the sample buffer used did not contain β-mercaptoethanol and DTT. All the uncropped and unprocessed gel images are provided in Fig. S9.

### Endotoxin detection assay

We measured the endotoxin level according to the Pierce™ Chromogenic Endotoxin Quant Kit. Briefly, 50 μl of different dilutions of endotoxin standard and test samples (1 mg/ml) were added to the plate which was pre equilibrated with heat at 37 °C. Next 50 μl of Amebocyte lysate reagent was added to each well and gently mixed. Further it was incubated for 30 min. Later, 100 μl of pre-warmed chromogenic substrate were added and incubated for 6 min followed by addition of 50 μl of stop solution (25% acetic acid) to each well and absorbance were measured at 405 nm. A standard curve was prepared from the absorbance of the endotoxin standard solutions. The endotoxin contaminations in each protein were calculated using the standard curve.

### Circular dichroism

Circular dichroism (CD) spectra were recorded on Jasco-1500 spectrophotometer in the wavelength ranging from 200 to 300 nm at 25 °C. A 1.0 ml sample (150 μg/ml) of each protein was loaded into a quartz cell. Minimum of three spectra for each sample were recorded, smoothed and corrected, and analyzed.

### Animals housing, immunization, and sample collection

All animal experiments were carried out as per the ethical approval (PRO/176/-MAY 2023, dt. 14.05.2020) accorded by the animal ethical committee of ICMR- National Institute of Cholera and Enteric Diseases (NICED), Kolkata, India. All the animals were taken from in house animal facility of NICED (registration number. 68/GO/ReBi/S/99/CPCSEA) and maintained at 25 °C and 45-55% humidity with 12-h alternate light and dark cycles. Immunogenicity was studied by intranasal administration of 60 µg of the recombinant proteins into female BALB/c mice (~5 weeks old) for three times at 12 days intervals. Mice were bled from the tail vein to collect blood on days 0, 11, 23, 35, 108, and 120 without sacrificing them. Stool was also collected prior to each immunization dose and 12 days after the last immunization dose. In a separate experiment, serum, stool, and intestinal wash were collected from the experimental mice twelve days after the last immunization. The mice were euthanized in a euthanasia chamber following the gradual carbon dioxide filling method from a compressed CO_2_ gas cylinder followed by decapitation as per the American Veterinary Medical Association (AVMA) guidelines approved by IAEC. Samples were collected and stored at −20 °C.

### Animal infection and protective efficacy

Four hours prior to the bacterial challenge of the immunized mice with *S*. Typhi Ty2, an iron-overload condition was created by intraperitoneal injection of Fe^3+^ as FeCl_3_ (SRL) in 10^−4^ N HCl (0.32 mg per gm of body weight) along with Desferoxamine (Novartis) (25 mg/kg body weight). Mice were orally challenged with 5 × 10^7^ *S*. Typhi bacteria and monitored for 10 days. In a separate experiment, mice from different immunized groups were subjected to an ileal loop experiment. To this end, mice were anaesthetized by intraperitoneal injection of a mixture of ketamine (35 mg kg^−1^ body weight; Sterfil Laboratories Pvt. Ltd, India) and xylazine (5 mg kg^−1^ body weight, AstraZeneca Pharma Ltd, India). A small abdominal incision was made and a loop (2–3 cm in length) in the distal ileum was created by suture. The closed ileal loop was instilled with 100 ng of cholera toxin (Sigma). After 8 h of the surgery, mice were euthanized by gradual carbon dioxide inhalation as described above. The loop weight/length ratio was quantified 8 h later.

### Enzyme linked immunosorbent assay (ELISA)

The binding affinity of the recombinant proteins, rCTB–T2544, rT2544, and rCTB for the GM1-ganglioside was measured by GM1-ELISA.To this end, ELISA plates were coated with 1 μg/well of GM1 ganglioside at 4 °C overnight. After washing of the wells with PBS-T (Phosphate buffer saline containing 0.05% Tween 20), blocking was done with 5% BSA in PBS for 1 h at 37 °C. Later on, the wells were incubated for 2 h at 37 °C with 100 µL of the recombinant proteins, serially diluted in PBS, and then washed again. Anti-His antibody (Cell Signaling Technology) (1:2500 dilution) was added to the plates and incubated for additional 1 h at 37 °C, followed by repeated washing with PBS-T. The wells were incubated with HRP-conjugated, goat anti-rabbit IgG for 1 h at 37 °C and developed by adding tetramethylbenzidine (TMB) (BD) substrate at room temperature. The absorbance was measured at 450 nm using a microplate reader.

To estimate antibody titers at days 0, 12, 24, 36, 108, 120, 96 well microtitre plates were coated with purified proteins (10 ng/well) overnight at 4 °C. After blocking the wells with 1% BSA in PBS to prevent nonspecific binding, they were incubated with the samples (serum, fecal extract, or intestinal wash contents) obtained from the immunized mice and serially diluted in PBS for 2 h. HRP-conjugated, goat anti-mouse IgG (1:10000) and goat anti-mouse IgA (1:5000) antibody was added to the respective wells and incubated for 1 h at 37 °C. The plates were developed using TMB substrate and the absorbance was measured using a microplate reader at 450 nm.

To test the avidity of IgG antibody against T2544, the sera (diluted 1:100) from CTB-T2544 and T2544 immunized mice after primary and booster immunization were allowed to react with T2544 coated wells for 2 h at 37 °C. The wells were washed three times for 5 min with PBS-Tween 20 with or without 4 M urea, followed by adding HRP-conjugated, goat anti-mouse IgG antibody to the wells and incubating for 1 h at 37 °C. The plates were developed using TMB substrate and the absorbance was measured using a microplate reader at 450 nm. The avidity index was represented as the ratio of the absorbance of the wells washed with and without 4 M Urea containing buffer and multiplying the value by 100.

### Enzyme-linked Immunospot (ELISpot) assay

Mixed cellulose ester membrane-bottom plates (Millipore) were coated with rT2544 or anti-mouse total immunoglobulin overnight at 4 °C. Wells were blocked with 1% BSA and incubated for 2 h at 37 °C. Cells isolated from mesenteric lymph nodes (MLN), Peyer’s Patches (PP), and spleen of immunized mice was added to blocked wells for 5 h, followed by washing with PBST. The plate was incubated with enzyme-conjugated anti-mouse IgG and IgA (Southern Biotech) overnight at 4 °C and developed with substrate. The number of spots was counted separately for each well. In a separate experiment, IFN-γ (ELISpot set from BD Bioscience) and IL-17 (ELISpot kit from R&D) pre coated mixed cellulose ester membrane-bottom plates were incubated with cells isolated from Peyer’s Patches (PP) stimulated with rT2544 for 24 h. The plate was incubated with enzyme-conjugated anti-mouse IFN-γ and IL-17 antibodies and developed with substrate. The number of spots was counted separately for each well.

### Bacterial adhesion inhibition assay

Bacterial cell suspensions were pre-incubated for 30 min with 1:50, 1:500, 1:5000 dilutions of the heat-inactivated immune serum, fecal extracts or intestinal contents. HT-29 Cell monolayers (5 × 10^5 cells/ml) were infected with bacteria at 1:10 M.O.I (multiplicity of infection) and synchronized by centrifugation at 400 × *g* for 5 min. Infected cells were incubated for 30 min and non-adhering bacteria were washed off with 1X PBS. Adherent bacteria were quantified by CFU counts after cell lysis with 1% triton-X100 and spreading over the lysates on LA agar plates containing Streptomycin 50 µg/ml. In a separate experiment, naïve mice were infected with or without bacteria pretreated with mucosal antibody present in fecal extract and intestinal wash. mice were euthanized by gradual carbon dioxide inhalation as described under “Methods” section on day 2, 4, 6 post-infection, and bacterial colonization was assessed in intestinal tissues and visceral organs.

### Opsonophagocytosis assay

Bacterial cell suspensions were pre-incubated for 30 min with 1:50 dilution of the heat inactivated immune serum, fecal extracts, or intestinal contents at 37 °C to allow for opsonization. The opsonized bacteria were then added to the THP-1 cell-derived macrophage monolayers (5 × 10^5 cells/ml), cultured in antibiotic-free complete medium at a multiplicity of infection of 1:10 (cell/bacteria) and centrifuged at 400 × *g* for 5 min to allow adhesion. The plates were incubated for 30 min at 37 °C. Following incubation, plates were washed three times with PBS. Extracellular bacteria were killed by incubating the plates with complete medium containing gentamicin (200 μg/ml) for 60 min. After washing the plates thrice with PBS, cells were lysed with 1% Triton X-100 for 15 min at 37 °C. The lysates were diluted in PBS and plated onto LA agar plates containing Streptomycin 50 μg/ml.

### Motility inhibition assay

For soft agar motility assay, 0.4% Bacto agar and 5% serum, fecal, or intestinal extracts were added to LB medium, which was poured on plates and allowed to settle at room temperature for 30 min. Bacterial culture was inoculated on the center of the plates and placed at 37 °C. The diameters of the concentrically growing bacterial cultures were measured for 6 h.

### RNA Isolation and quantitative real-time PCR

Dendritic cells (DCs) were separated from the other cells of the MLN of the PBS-immunized and experimental immunized mice using CD11c^+^ magnetic beads (Miltenyi Biotech). Total RNA was extracted from the DCs, and cDNA was prepared using a cDNA synthesis kit according to the manufacturer’s instructions. Transcript levels were determined by quantitative real-time PCR, using SYBR Green PCR Master Mix (Applied Biosystem) on StepOnePlus real time PCR system (Applied Biosystem). GAPDH levels were taken for normalization, fold changes were calculated using 2^−ΔΔCt^. In a separate experiment, CTB-T2544 and PBS immunized mice were euthanized by gradual carbon dioxide inhalation as described under *Methods* section after 12 days of three successful immunization and CD11c^int^MHCII^hi^CD103^+^ cells were stained with fluorochrome-tagged antibody and evaluated using flow cytometer.

### Flow cytometry

Twelve days after the last immunization, the PBS and experimental immunized mice were euthanized by gradual carbon dioxide inhalation as described under Methods section. B and T cell subsets were isolated and stained with fluorochrome-conjugated antibodies (BD Biosciences) against specific surface markers. After staining, the cells were washed and analyzed by flow cytometry (BD FACS ARIA II) following standard protocol. The gating strategy is provided in Fig. S8. All the used antibodies are listed in Supplementary Table 1 (Table S1).

### Memory T cell assay

Dendritic cells were generated from bone marrow (BM) cells collected from the femurs and tibias of BALB/c mice. Briefly, BM cells, after collection, were cultured in 90 mm dish in complete RPMI 1640 medium supplemented with murine recombinant GM-CSF (20 ng/ml). Sixty percent of the culture medium was replaced every 3rd day with fresh medium. Cells were collected on day 7 and starved by culturing in RPMI 1640 containing 1% FBS for 12 h, before treatment with rT2544 for 24 h. To generate memory T cells, immunized mice were euthanized by gradual carbon dioxide inhalation as described under *Methods* section. CD4^+^ T lymphocytes were isolated from the immunized mice after 120 days and co-cultured for 5 days with antigen-pulsed BMDCs at 37 °C and in the presence of 5% CO_2_. Memory T cells (CD4^+^CD62L^low^CD44^hi^, CD4^+^CD62L^hi^CD44^hi^) were studied by flow cytometry and the culture supernatants were assayed for IFN-γ by ELISA. All the used ELISA and ELISPOT kits are listed in the Supplementary Table 2 (Table S2).

### Statistical analyses

Statistical analyses of data were performed using GraphPad Prism 8.0.1.244. Log rank Mantel-Cox test was performed to analyze survival curves. Unpaired *t* test was used to compare between two groups, while one-way and two-way ANOVA with *post hoc* Tukey’s multiple comparison tests were performed to compare amongst more than two groups. A *P* value of <0.05 was considered significant; *p: 0.3332, **p: 0.0021 ***p: 0.0002, ****p < 0.0001. Data represented as mean ± SD. Error bars represent SD.

### Reporting summary

Further information on research design is available in the Nature Research Reporting Summary linked to this article.

### Supplementary information

Supplemental Material

Reporting Summary

## Data Availability

Data will be made available upon request from the corresponding author (Santasabuj Das; email id- santasabujdas@yahoo.com).
