# Supplementary material for: Intranasal immunization of mice with chimera of Salmonella Typhi protein elicits protective intestinal immunity
Source: NPJ Vaccines. 2024 Feb 6;9:24. doi: 10.1038/s41541-024-00812-4 (PMC10847434; doi:10.1038/s41541-024-00812-4)
Supplement: Supplementary file 1 — Supplemental Material [file 41541_2024_812_MOESM1_ESM.pdf]

Intranasal Immunization of Mice with a Chimeric Antigen of Cholera Toxin B and *Salmonella* Typhi outer membrane protein T2544 elicits protective antibodies and T cell response at the intestinal mucosa

Suparna Chakraborty [a], Pujarini Dutta [a, b], Ananda Pal [a], Swarnali Chakraborty [a], George Banik [c], Prolay Halder [d], Animesh Gope [a], Shin-ichi Miyoshi [e,f], Santasabuj Das [a, g *]

a Division of Clinical Medicine, ICMR- National Institute of Cholera and Enteric Diseases, P-33, C.I.T. Road, Scheme XM, Beliaghata, Kolkata-700 010, India

b Department of Pediatrics, Steele Children's Research Center, University of Arizona, Tuscon, Arizona.

c BD Biosciences, INDIA, Smart works Business Center, Victoria Park, 37/2 GN Block, Sector 5, Saltlake City, Kolkata-700091

d Division of Bacteriology, ICMR- National Institute of Cholera and Enteric Diseases, P-33, C.I.T. Road, Scheme XM, Beliaghata, Kolkata-700 010, India.

e Graduate School of Medicine, Dentistry and Pharmaceutical Sciences, Okayama University, Okayama Japan.

f Collaborative Research Center of Okayama University for Infectious Diseases at Indian Council of Medical Research- National Institute of Cholera and Enteric Diseases, Kolkata, 700010, India

g ICMR-National Institute of Occupational Health, Meghaninagar, Ahmedabad, Gujrat-3800016.

[*]Corresponding author. Email: santasabujdas@yahoo.com

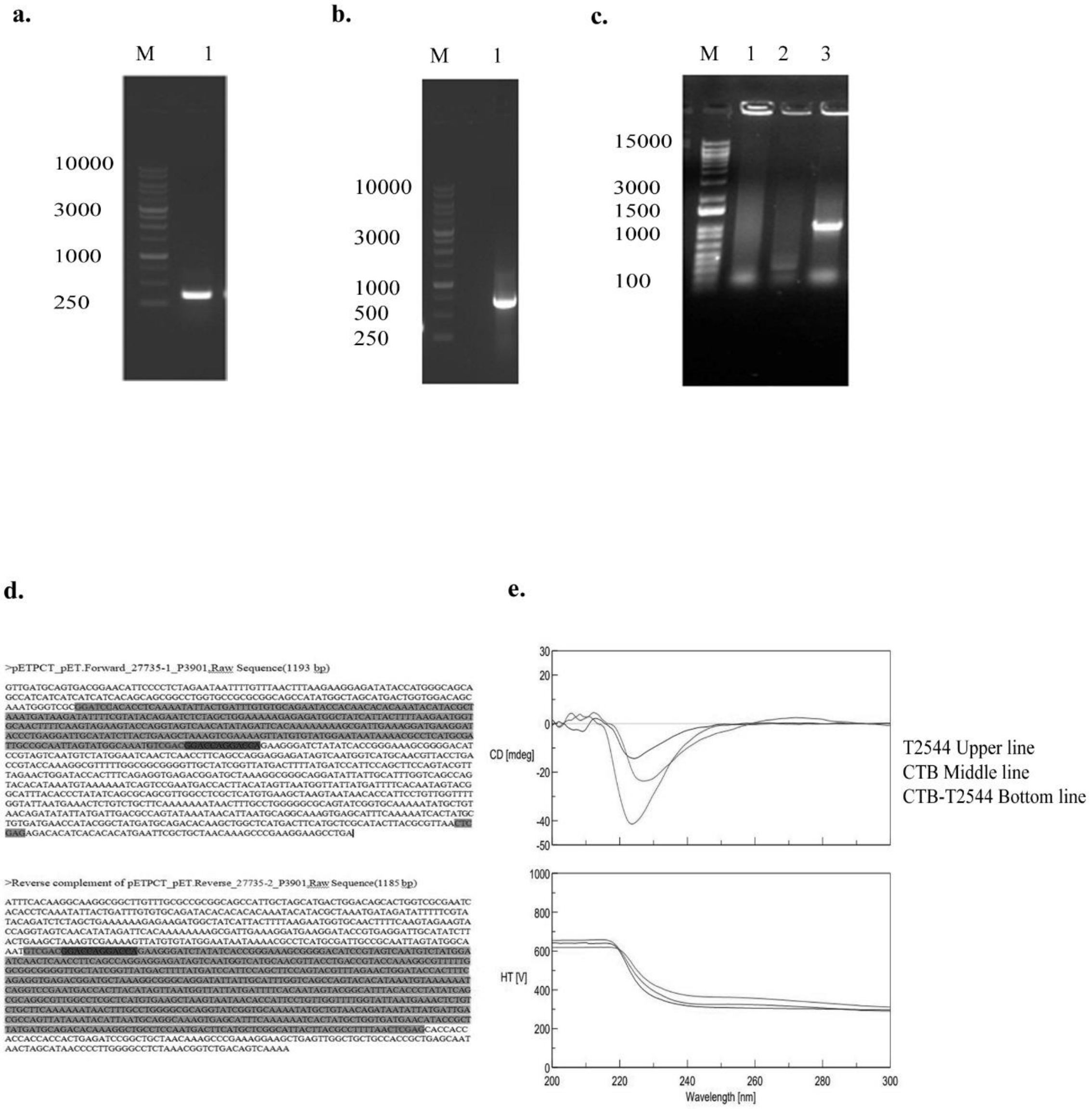

Supplementary Figure S1 (FIG. S1). Production and characterization of CTB-T2544. (a) PCR amplification of cholera toxin B (ctxB) gene; Lane-M, 1Kb plus DNA ladder. (b) PCR amplification of t2544 gene along with GPGP linker; Lane-M, 1Kb plus DNA ladder. (c) Screening for positive clones of ctb-t2544 by PCR amplification after ligation into the pET28a vector, Lane-M, 1 Kb plus DNA ladder; Lane 1-3, ligated clones. All the agarose gel images are derived from the same experiment and were processed in parallel. (d) Nucleotide

sequencing of CTB and T2544 in pET-ctb-t2544. (e) Far UV CD spectral analysis of purified recombinant proteins were recorded at 25°C and virtually superimposed. High tension (HT) voltage readings of the three proteins, recorded simultaneously showed no noise.

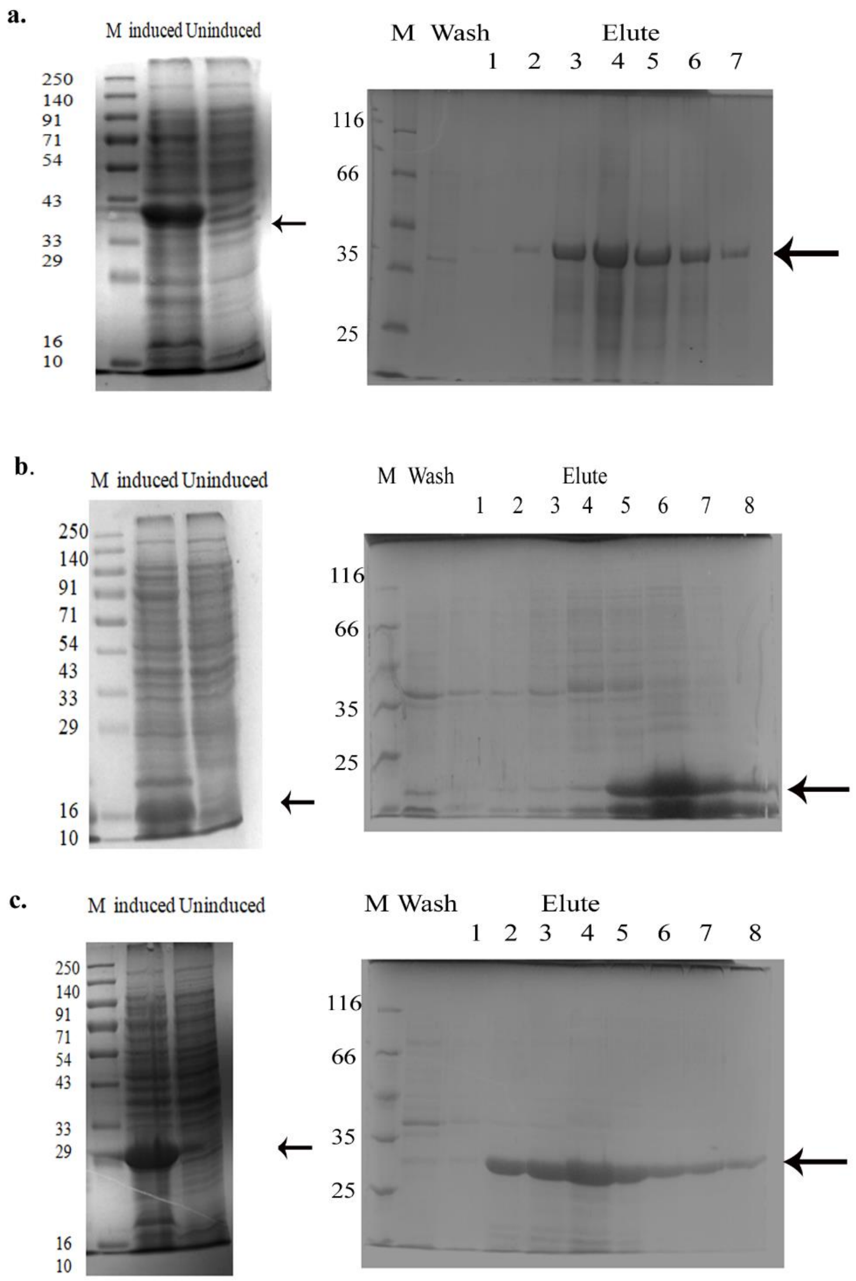

Supplementary Figure S2 (FIG. S2). Recombinant protein expression and purification. a-c (left panel). SDS-PAGE profile of crude cell lysates showing recombinant protein expression induced by IPTG (1mM). Lane M, Pre-stained protein marker; Lane Induced, cell lysates after protein induction by IPTG; Lane Uninduced, cell lysates of parallel culture without protein induction. Arrows indicate the respective induced proteins. (Right panel) SDS-PAGE profile

of protein purified by Ni-column chromatography. Lane-M, Protein marker; Lane wash – flow through of column wash, Lane 1-8 - eluted fractions of purified proteins; Arrow indicates the respective purified proteins. a, b and c represent CTB-T2544, CTB and T2544, respectively. All the agarose gel images are derived from the same experiment and were processed in parallel.

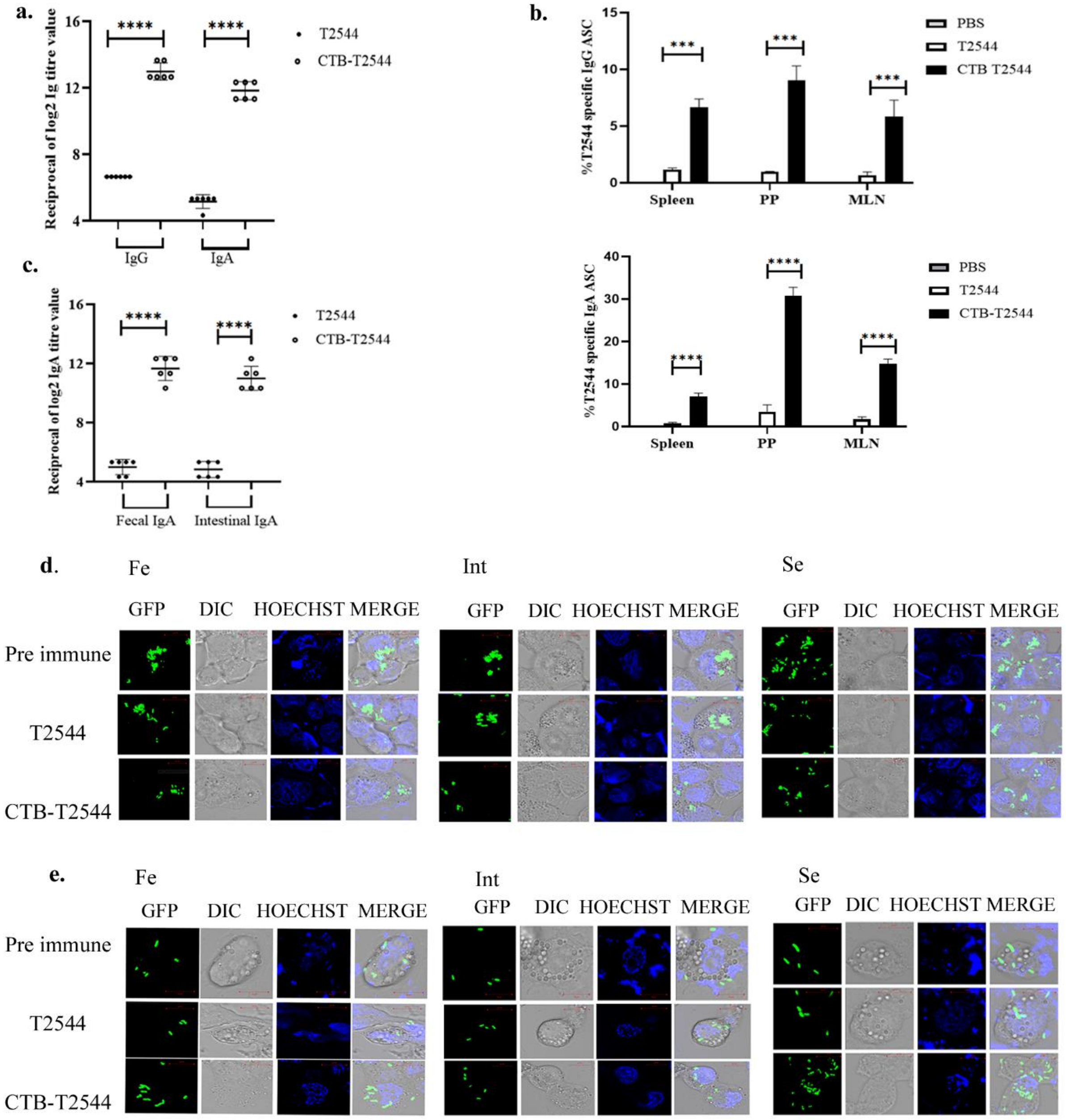

Supplementary Figure S3 (FIG. S3). Antibodies and their functionality. (a,b) CTB-specific antibodies determined by ELISA in the serum (a) and feces and intestinal wash (b) of the immunized mice (n=6). (c) Spots counted in the experiment described under figure 3b were plotted as percentages of T2544-specific IgG and IgA ASCs out of the respective total ASCs. Significance was calculated with respect to similar cell populations from PBS-immunized mice using two tailed unpaired t test. Error bars represent SD. ****p<0.0001, *** p<0.0002 (d) Cell

adhesion assay. *Salmonella* Typhi Ty2 expressing green fluorescent protein (appearing green) were pre-incubated for 30 mins with the serum, feces and intestinal lavage from different immunized groups and used for infection of HT-29 cell monolayer. Nucleus of the cell was stained with Hoechst (appearing blue) and the cells were viewed under confocal microscope. Scale bar =10 µm. (e) Opsonophagoctosis assay. GFP-expressing *S*. Typhi Ty2 (appearing green) were pre-incubated with serum and fecal extracts or intestinal lavage. THP-1 cell monolayers were used for infection with the opsonized bacteria for 1h, followed by gentamicin protection assay. Nucleus of the cell was stained with Hoechst (appearing blue), followed by viewing under confocal microscope. Scale bar =10 µm.

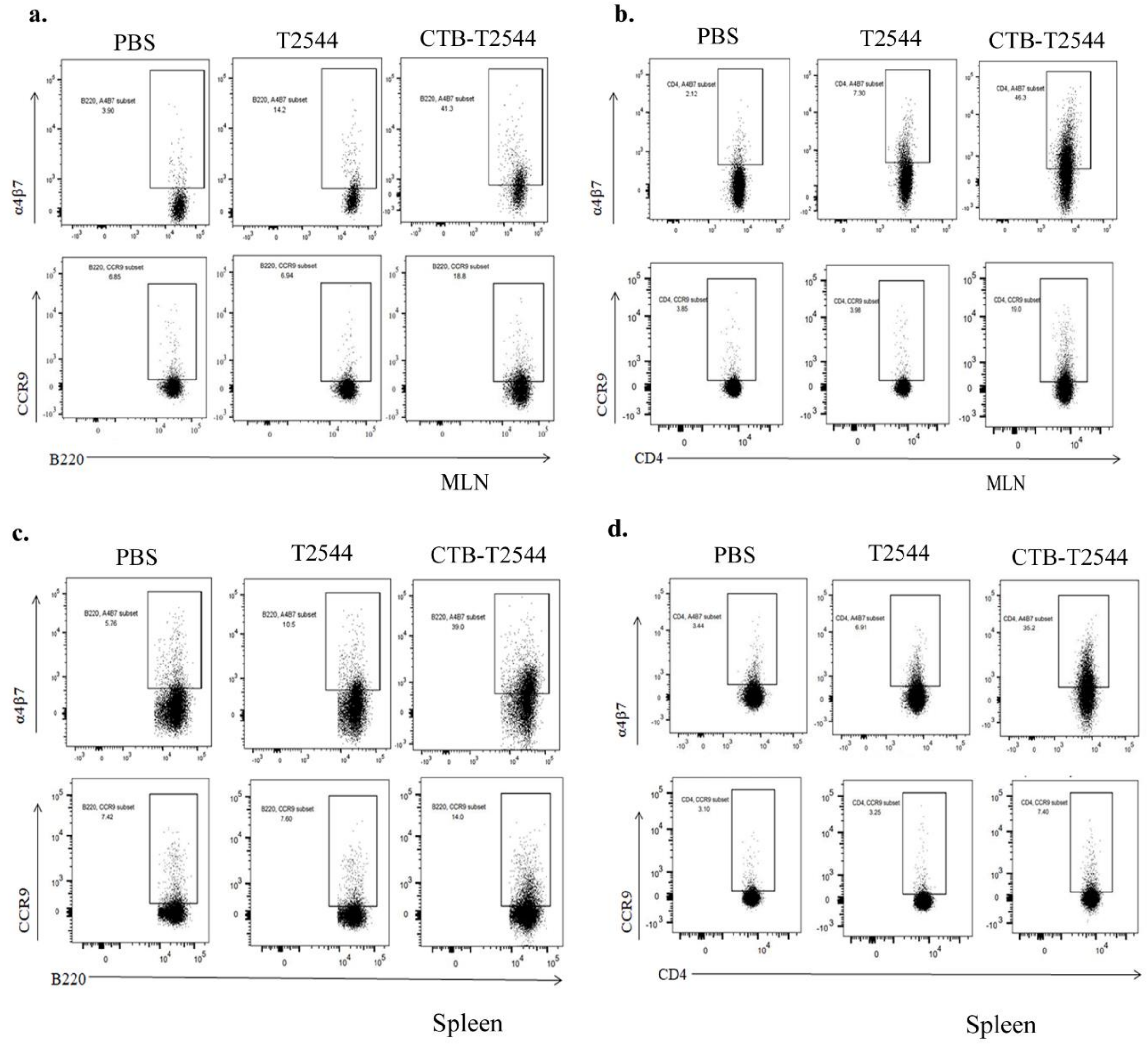

Supplementary Figure S4 (FIG. S4). Gut homing receptor expression by B and T cells. Homing receptor expression on lymphocytes from the MLN (a,b) and spleen (c,d) were quantified by flow cytometry. Data were analyzed using FlowJo software.

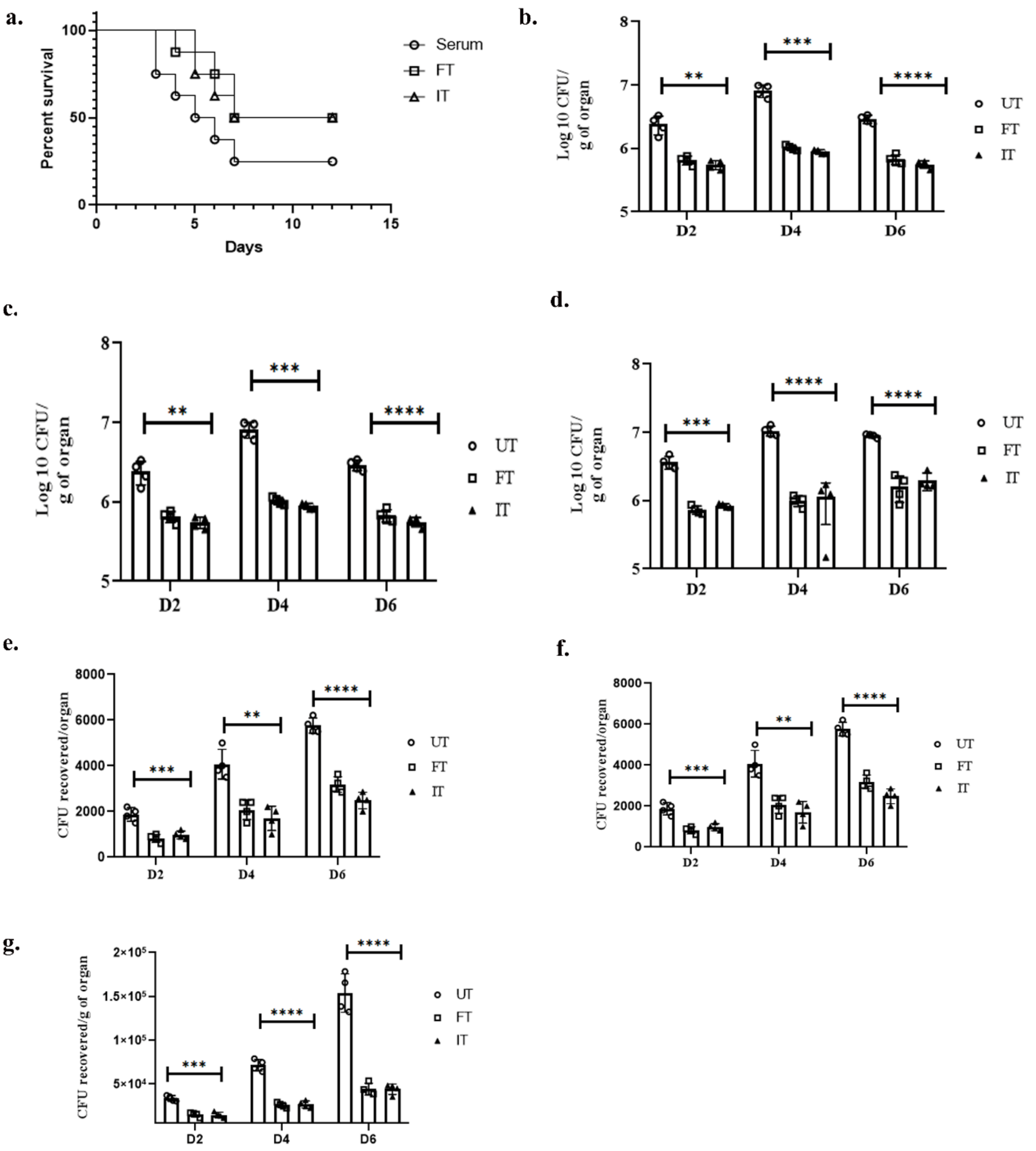

Supplementary Figure S5 (Fig. S5). **CTB-T2544 immunization induces protection from *S.* Typhi challenge**. a. Pooled sera from rCTB-T2544-immunized BALB/c mice (n = 8) were adoptively transferred to naïve mice (n=8), followed by challenge with 5×10^6 CFU of *S.* Typhi through orogastric route. Similar experiments were performed in parallel by infecting naïve mice (n=8/group) with *S.* Typhi, pre-incubated with fecal extracts or intestinal lavage from rCTB-T2544 immunized mice. Animals were monitored for the next 12 days and percent death

was calculated. b-g. In separate experiments, three groups of naive BALB/c mice (n= 12/groups; UT, FT and IT) were challenged with 10^4 CFU of *S*. Typhi Ty2, pre-incubated with or without fecal extracts and intestinal lavage from CTB-T2544 immunized mice. Mice were euthanized at days 2, 4, and 6. Bacterial load in the intestinal tissues (Ileum b, Cecum c colon d), Mesenteric lymph nodes (MLN, e) and visceral organs (Spleen f and liver g) were determined by culturing organ homogenates on LB Agar plates with streptomycin selection (50 µg/ml). Significance was calculated by comparing recovered CFU of bacteria from FT and IT group with UT group using unpaired t tests. UT- untreated bacteria, FT- bacteria treated with fecal extracts, IT- bacteria treated with intestinal lavage samples. Two-tailed unpaired t tests were performed. ****p<0.0001, ***p<0.0002, **p: 0.0021, *p : 0.3332. Error bars represent SD.

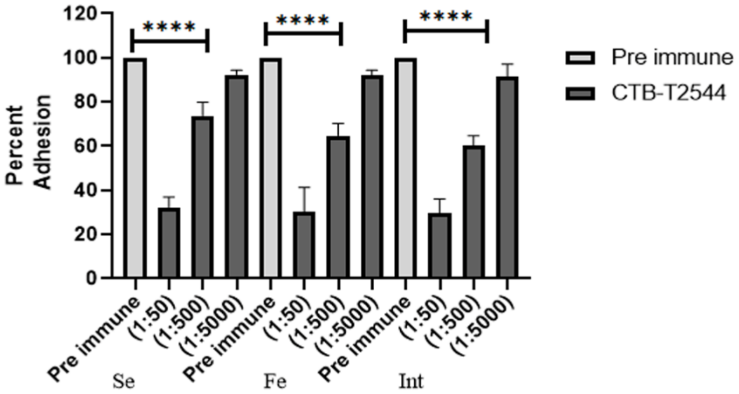

Supplementary Figure S6 (Fig S6). **Mucosal antibody pretreatment prevents percent adhesion of bacteria in HT-29 cells.** Bacteria were pre-incubated with different dilutions of the serum and secretory antibodies for 30 min, followed by infection of HT-29 cells. Bacterial adhesion was calculated by CFU counts, recovered from the infected cells after overnight culture on Luria Agar plates, containing streptomycin (50 µg/ml). Percent adhesion for CTB-T2544 immunized samples was calculated with respect to adhesion for the corresponding pre-immune samples, considering it as 100 percent. Significance was calculated using unpaired t test between CTB-T2544 and Preimmune sample. ****$p<0.0001$. Error bars represent SD.

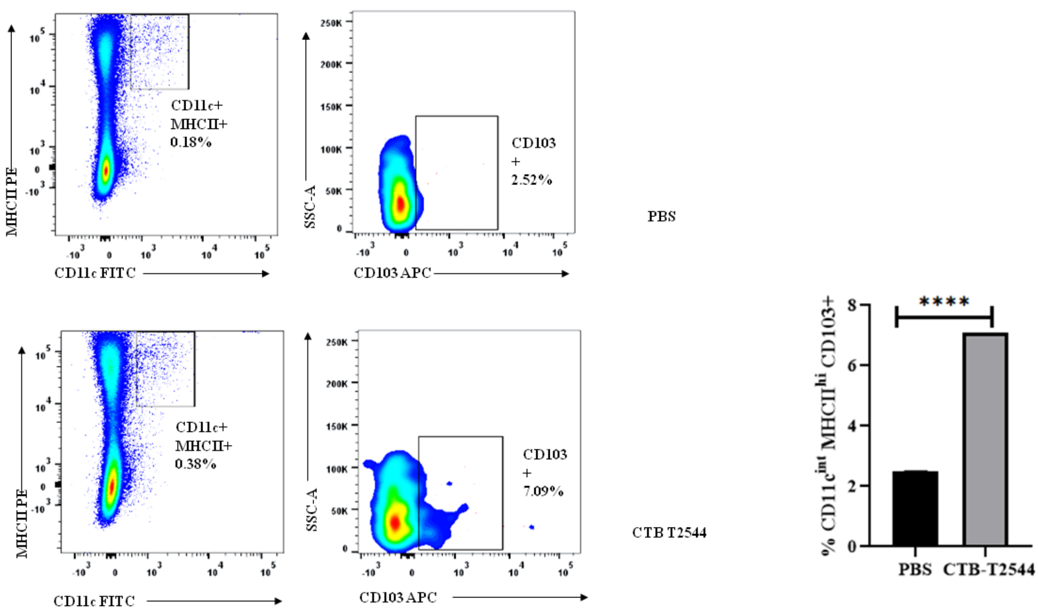

Supplementary Figure S7 (Fig S7). Intranasal CTB-T2544 promotes CD103+ DCs migration to the MLN. MLN cells were isolated from CTB-T2544 and PBS immunized mice and number of CD103+ cells were quantified in the cell population gated for CD11c^{int} and MHCII^{hi} expression. Significance was calculated using unpaired t test between CTB-T2544 and PBS groups. ****p<0.0001. Error bars represent SD. Representative flow images are provided here.

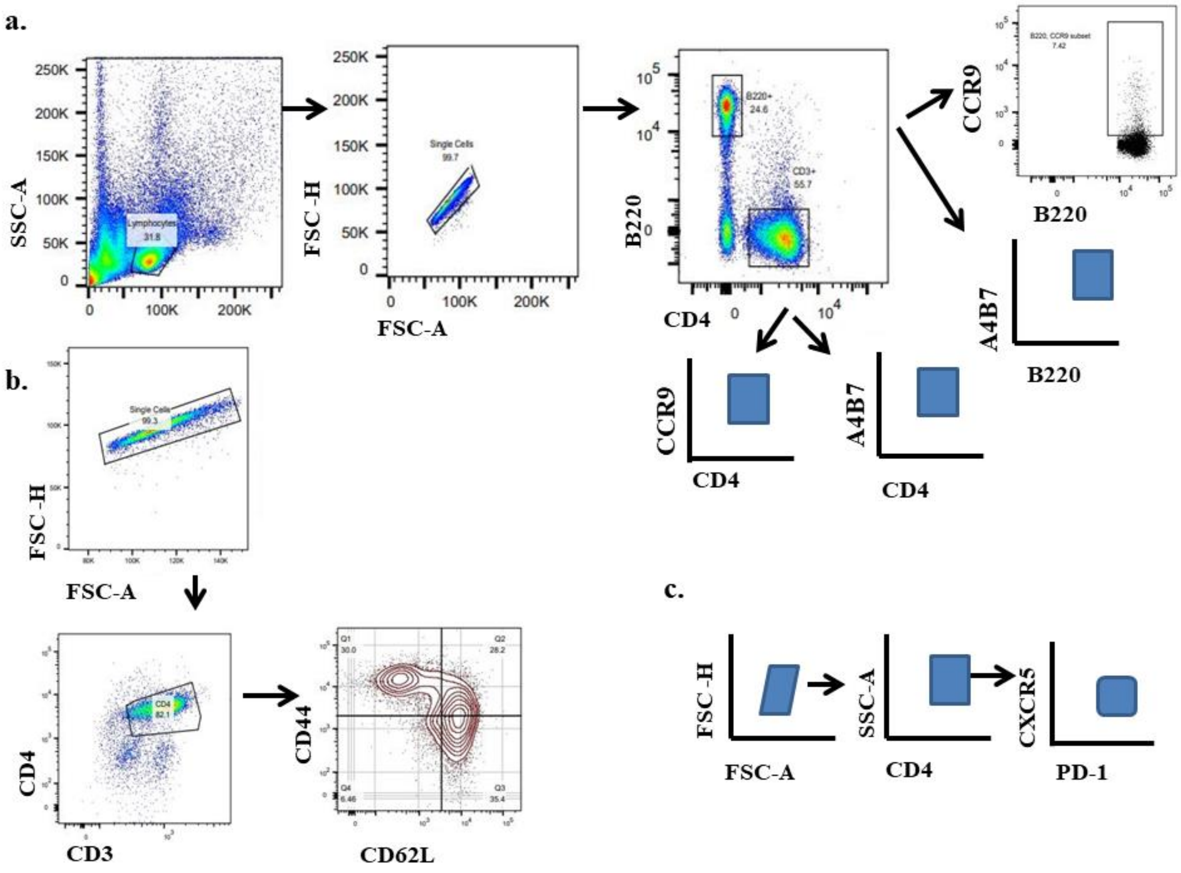

Supplementary Figure S8 (Fig. S8). Gating Strategy. a. Gating strategy for gut homing analysis from spleenocytes and MLN. b. Gating strategy for memory T cell analysis. c. Pictorial gating scheme for Follicular helper T cell (T_{FH}) analysis.

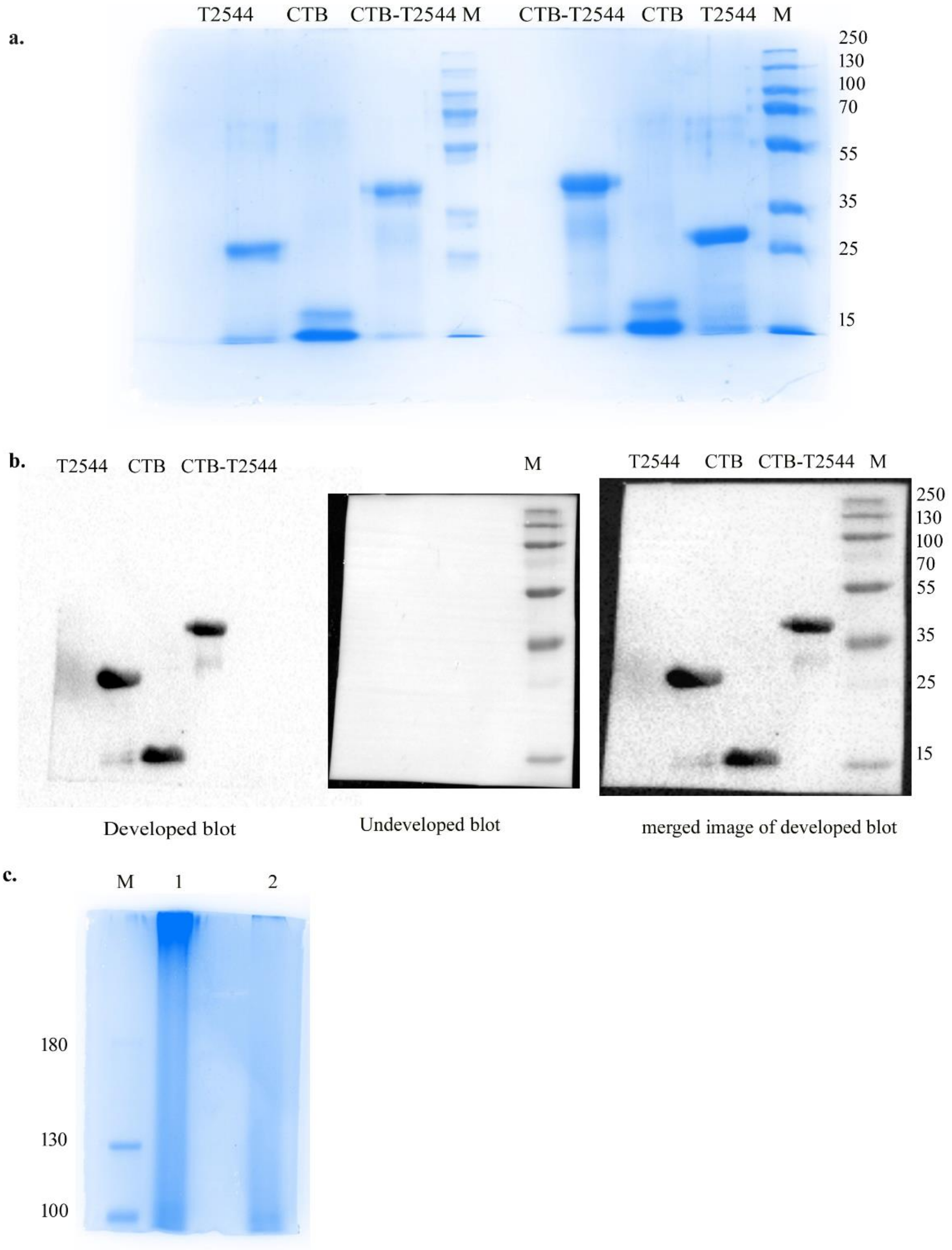

Supplementary Figure S9 (Fig. S9). Uncropped and unprocessed gel and blot images. a. Three recombinant proteins T2544, CTB and CTB-T2544 run on 10% SDS PAGE, followed by Coomassie blue staining, Lane-M, Pre stained protein marke; b. Western blot analysis of purified recombinant proteins; the blot was developed using anti-HIS monoclonal antibody; Lane-M, Pre-stained protein marker. (c) Denatured (boiled) and non-denatured (unboiled) rCTB-T2544 protein resolved in 10% SDS PAGE, followed by Coomassie blue staining of the

gel. Lane – M, Pre-stained protein marker, Lane 1- unboiled rCTB-T2544; Lane 2- rCTB-T2544 after boiling.

Supplementary Table 1.Table S1. Antibody list. All the antibodies used, are listed.

ANTIBODY (flow cytometry)				
Antigen (mouse)	Flurochrome	Origin	Cat #	Manufacturer
CD4	APC H7	Rat	560181	BD biosciences
CD4	PE	Rat	553048	BD biosciences
CD3	perCPcy5.5	Rat	560527	BD biosciences
B220	PeCy7	Rat	552772	BD biosciences
CD11C	FITC		557400	BD biosciences
MHC II (I-A^{d})	BIOTIN-<br>STREPTAVIDIN PE	Mouse	553546<br>554061	BD biosciences<br>BD biosciences
CD103	APC	Rat	562772	BD biosciences
A4B7 (LPAM)	PE CF594	RAT	562668	BD biosciences
CCR9 (CD199)	PE	Mouse	565576	BD biosciences
PD1 (CD279)	PE	hamster	561788	BD biosciences
CxCR5	FITC	RAT	561989	BD biosciences
CD44	APC	Rat	561862	BD biosciences
CD62L	PeCy7	Rat	560516	BD biosciences

ANTIBODY (western blot)				
Antigen (mouse)	Flurochrome	Origin	Cat #	Manufacturer
HIS-tag (western blot)	Nil	Rabbit	2365S	Cell Signaling Technology
ANTIBODY (ELISA)				
Antigen (mouse)	Flurochrome	Origin	Cat #	Manufacturer
Anti mouse IgG - HRP		Goat	31432	Thermo
Anti rabbit IgG –HRP		Goat	31464	Thermo
Anti mouse IgA- HRP		Goat	A4789-1ML	Sigma
ANTIBODY (ELISPOT)				
Antigen (mouse)	Flurochrome	Origin	Cat #	Manufacturer
IgG-Hrp (human adsorbed)	HRP	Goat	1030-05	Southern biotech
IgA-AP	AP	Goat	1040-04	Southern biotech

Supplementary Table 2. Table S2. ELISA and ELISPOT kit. All the ELISA and ELISPOT kits used, are listed.

ELISA kit (mouse)		
Kit name	Cat #	Manufacturer
IL2	555148	BD Biosciences
IL-4	555232	BD Biosciences
IL-5	555236	BD Biosciences
IL-6	555240	BD Biosciences
IL-10	555252	BD Biosciences
IL-12	555256	BD Biosciences
IFNγ	DY485-05	BD Biosciences
TNFA	558534	BD Biosciences
ELISPOT kit (mouse)		
Kit name	Cat #	Manufacturer
IFNγ	EL485	R&D
IL-17	EL421	R&D
