# Supplementary material for: Intranasal immunization of mice with chimera of Salmonella Typhi protein elicits protective intestinal immunity
Source: NPJ Vaccines. 2024 Feb 6;9:24. doi: 10.1038/s41541-024-00812-4 (PMC10847434; doi:10.1038/s41541-024-00812-4)
Supplement: Supplementary file 2 — Reporting Summary [file 41541_2024_812_MOESM2_ESM.pdf]

nature portfolio

Corresponding author(s): Santasabuj Das

Last updated by author(s): 01/21/2024

Reporting Summary

Nature Portfolio wishes to improve the reproducibility of the work that we publish. This form provides structure for consistency and transparency in reporting. For further information on Nature Portfolio policies, see our Editorial Policies and the Editorial Policy Checklist.

Statistics

For all statistical analyses, confirm that the following items are present in the figure legend, table legend, main text, or Methods section.

n/a	Confirmed	
☐	☒	The exact sample size (*n*) for each experimental group/condition, given as a discrete number and unit of measurement
☐	☒	A statement on whether measurements were taken from distinct samples or whether the same sample was measured repeatedly
☐	☒	The statistical test(s) used AND whether they are one- or two-sided<br>*Only common tests should be described solely by name; describe more complex techniques in the Methods section.*
☒	☐	A description of all covariates tested
☒	☐	A description of any assumptions or corrections, such as tests of normality and adjustment for multiple comparisons
☐	☒	A full description of the statistical parameters including central tendency (e.g. means) or other basic estimates (e.g. regression coefficient) AND variation (e.g. standard deviation) or associated estimates of uncertainty (e.g. confidence intervals)
☐	☒	For null hypothesis testing, the test statistic (e.g. *F*, *t*, *r*) with confidence intervals, effect sizes, degrees of freedom and *P* value noted<br>*Give P values as exact values whenever suitable.*
☒	☐	For Bayesian analysis, information on the choice of priors and Markov chain Monte Carlo settings
☒	☐	For hierarchical and complex designs, identification of the appropriate level for tests and full reporting of outcomes
☒	☐	Estimates of effect sizes (e.g. Cohen's *d*, Pearson's *r*), indicating how they were calculated

Our web collection on statistics for biologists contains articles on many of the points above.

Software and code

Policy information about availability of computer code

Data collection	K2D2 (http://cbdm-01.zdv.uni-mainz.de/~andrade/k2d2/) was used for secondary structure prediction.
Data analysis	Statistical analyses of data were performed using GraphPad Prism 8.0.1.244. Log rank Mantel- Cox test was performed to analyze survival curves. Unpaired t-test was used to compare between two groups while one way and two-way ANOVA with post hoc Tukey's multiple comparison tests were performed to compare amongst more than two groups. A P value of < 0.05 was considered significant; *p: 0.3332, **p: 0.0021 ***p: 0.0002, ****p < 0.0001. Data represented as mean ± SD. Error bars represent SD.

For manuscripts utilizing custom algorithms or software that are central to the research but not yet described in published literature, software must be made available to editors and reviewers. We strongly encourage code deposition in a community repository (e.g. GitHub). See the Nature Portfolio guidelines for submitting code & software for further information.

Data

Policy information about availability of data

All manuscripts must include a data availability statement. This statement should provide the following information, where applicable:

- Accession codes, unique identifiers, or web links for publicly available datasets
- A description of any restrictions on data availability
- For clinical datasets or third party data, please ensure that the statement adheres to our policy

Data will be available upon request.

Research involving human participants, their data, or biological material

Policy information about studies with human participants or human data. See also policy information about sex, gender (identity/presentation), and sexual orientation and race, ethnicity and racism.

Reporting on sex and gender	NA
Reporting on race, ethnicity, or other socially relevant groupings	NA
Population characteristics	NA
Recruitment	NA
Ethics oversight	NA

Note that full information on the approval of the study protocol must also be provided in the manuscript.

Field-specific reporting

Please select the one below that is the best fit for your research. If you are not sure, read the appropriate sections before making your selection.

☒ Life sciences ☐ Behavioural & social sciences ☐ Ecological, evolutionary & environmental sciences

For a reference copy of the document with all sections, see nature.com/documents/nr-reporting-summary-flat.pdf

Life sciences study design

All studies must disclose on these points even when the disclosure is negative.

Sample size	For different experiments, each groups contains 4,6 or 10 mice and clearly mentioned.
Data exclusions	No data were excluded
Replication	Experiments were done with biological and technical triplicates.
Randomization	All the animals were taken from the in-house animal facility of NICED and randomly divided into different experimental groups.
Blinding	NA

Reporting for specific materials, systems and methods

We require information from authors about some types of materials, experimental systems and methods used in many studies. Here, indicate whether each material, system or method listed is relevant to your study. If you are not sure if a list item applies to your research, read the appropriate section before selecting a response.

Materials & experimental systems

n/a	Involved in the study	
☐	☒	Antibodies
☐	☒	Eukaryotic cell lines
☒	☐	Palaeontology and archaeology
☐	☒	Animals and other organisms
☒	☐	Clinical data
☒	☐	Dual use research of concern
☒	☐	Plants

Methods

n/a	Involved in the study	
☒	☐	ChIP-seq
☐	☒	Flow cytometry
☒	☐	MRI-based neuroimaging

Antibodies

Antibodies used	HRP-conjugated anti-rabbit IgG and anti-mouse IgG were purchased from Thermo. All the antibodies used in flowcytometric experiments were procured from BD biosciences. Anti mouse IgA -AP and anti mouse IgG - HRP were purchased from Sourthen Biotech. Anti mouse IgA-HRP was purchased from sigma (All antibodies are enlisted in a separate Supplementary Table 1)
Validation	All the antibodies were used by our lab in the previous studies and are also being used for ongoing studies and by other researchers in the field.

Eukaryotic cell lines

Policy information about cell lines and Sex and Gender in Research

Cell line source(s)	THP-1 and HT-29 cells lines were purchased from ATCC . Bone marrow derived Dendritic cells were collected from the femurs and tibias of BALB/c mice.
Authentication	THP-1 and HT-29 cells were purchased from ATCC and thus authenticated by the company. Dendritic cells were generated from the mouse bone marrow (BM) cells and the authentication was confirmed by surface antigen expression, as measured by flow cytometry
Mycoplasma contamination	Mycoplasma contamination was ruled out by performing Mycoplasma specific PCR .
Commonly misidentified lines (See ICLAC register)	NA

Animals and other research organisms

Policy information about studies involving animals; ARRIVE guidelines recommended for reporting animal research, and Sex and Gender in Research

Laboratory animals	All the Balb/c mice were taken from in house animal facility of NICED (registration number. 68/GO/ReBi/S/99/CPCSEA)
Wild animals	NA
Reporting on sex	Only female mice were used.
Field-collected samples	NA
Ethics oversight	All animal experiments were carried out as per the ethical approval (PRO/176/ -MAY 2023, dt. 14.05.2020) accorded by the animal ethical committee of ICMR- National Institute of Cholera and Enteric Diseases (NICED), Kolkata, India.

Note that full information on the approval of the study protocol must also be provided in the manuscript.

Plants

Seed stocks	*Report on the source of all seed stocks or other plant material used. If applicable, state the seed stock centre and catalogue number. If plant specimens were collected from the field, describe the collection location, date and sampling procedures.*
Novel plant genotypes	*Describe the methods by which all novel plant genotypes were produced. This includes those generated by transgenic approaches, gene editing, chemical/radiation-based mutagenesis and hybridization. For transgenic lines, describe the transformation method, the number of independent lines analyzed and the generation upon which experiments were performed. For gene-edited lines, describe the editor used, the endogenous sequence targeted for editing, the targeting guide RNA sequence (if applicable) and how the editor was applied.*
Authentication	*Describe any authentication procedures for each seed stock used or novel genotype generated. Describe any experiments used to assess the effect of a mutation and, where applicable, how potential secondary effects (e.g. second site T-DNA insertions, mosiacism, off-target gene editing) were examined.*

Flow Cytometry

Plots

Confirm that:

☒ The axis labels state the marker and fluorochrome used (e.g. CD4-FITC).

☒ The axis scales are clearly visible. Include numbers along axes only for bottom left plot of group (a 'group' is an analysis of identical markers).

☒ All plots are contour plots with outliers or pseudocolor plots.

☒ A numerical value for number of cells or percentage (with statistics) is provided.

Methodology

Sample preparation	Sample were prepared as per guidelines provided by the BD biosciences.
Instrument	BD FACS aria II
Software	Flow Jo (BD Biosciences)
Cell population abundance	Abundance was measured by surface antibody staining of the respective positive and negative cell markers as per experiment. Fluorescence minus one method was used while staining a single tube with various markers needed.
Gating strategy	Gating strategy is provided yet.

☒ Tick this box to confirm that a figure exemplifying the gating strategy is provided in the Supplementary Information.
